# Pharmacologic modulation of 5-fluorouracil by folinic acid and pyridoxine for treatment of patients with advanced breast carcinoma

**DOI:** 10.1038/s41598-022-12998-5

**Published:** 2022-05-31

**Authors:** David Machover, Emma Goldschmidt, Wathek Almohamad, Vincent Castagné, Julien Dairou, Christophe Desterke, Léa Gomez, Yann Gaston-Mathé, Claude Boucheix

**Affiliations:** 1 INSERM U935-UA09 and Institut de Cancérologie et d’Immunogénétique (ICIG), Paul-Brousse Hospital, University Paris-Saclay, 12, Avenue Paul-Vaillant-Couturier, 94800 Villejuif, France; 2 Department of Medical Oncology, Paul-Brousse Hospital, Assistance Publique-Hôpitaux de Paris (APHP), University Paris-Saclay, 94800 Villejuif, France; 3 Department of Pharmacy, Paul-Brousse Hospital, APHP, University Paris-Saclay, 94800 Villejuif, France; 4 Laboratory of Pharmacologic Biochemistry and Toxicology, CNRS UMR 8601, University Paris-Descartes, 45, Rue des Saints-Pères, 75006 Paris, France; 5 Department of Biophysics and Nuclear Medicine, Kremlin-Bicêtre Hospital, APHP, University Paris-Saclay, 94270 Le Kremlin-Bicêtre, France; 6 YGM Consult SAS, 75015 Paris, France

**Keywords:** Cancer, Drug discovery, Oncology

## Abstract

High concentration pyridoxal 5’-phosphate, the cofactor of vitamin B6, potentiates cytotoxicity in cancer cells exposed to 5-fluorouracil (FUra) and folinic acid (FA). We studied the effect of high-dose pyridoxine on antitumor activity of regimens comprising FUra and FA in 27 advanced breast carcinoma patients. Of 18 previously untreated patients, 12 had tumors that did not overexpress HER2 (Group I), and 6 that overexpressed HER2 (Group II). Nine patients (Group III) had prior chemotherapy. Group I received *AVCF* (doxorubicin, vinorelbine, cyclophosphamide, FUra, FA) or *FAC* (doxorubicin, cyclophosphamide, FUra, FA) followed by *TCbF* (paclitaxel carboplatin, FUra, FA). Groups II, and III received *TCbF*. Pyridoxine iv (1000–3000 mg/day) preceded each FA and FUra. Group II also received trastuzumab and pertuzumab. 26 patients responded. Three patients in Group I had CRs and 9 had PRs with 62–98% reduction rates; 4 patients in Group II had CRs and 2 had PRs with 98% reduction. Of 7 measurable patients in Group III, 2 attained CRs, and 5 had PRs with 81–94% reduction rates. Median time to response was 3.4 months. Unexpected toxicity did not occur. This pilot study suggests that high-dose vitamin B6 enhances antitumor potency of regimens comprising FUra and FA.

## Introduction

Fluorodeoxyuridine monophosphate (FdUMP), the active metabolite of 5-fluorouracil (FUra), binds to thymidylate synthase (TS) and the folate cofactor 5,10-methylene tetra hydro pteroylglutamate (CH_2_-H_4_PteGlu) to form a TS-inactivating [FdUMP-TS-CH_2_-H_4_PteGlu] ternary complex, whose dissociation decreases as CH_2_-H_4_PteGlu is augmented over a wide concentration range up to levels greater than 1 mM^1–3^. Exposure of cancer cells to 5-fluorodeoxyuridine with high concentration 5-formyl tetra hydro pteroylglutamate [5-HCO-H_4_PteGlu; folinic acid (FA); leucovorin] up to 20 µM in vitro resulted in formation of greater amounts of ternary complex than with the single fluoropyrimidine leading to gradual enhancement of the cytotoxic effect^4^. FdUMP-mediated TS inhibition prevents synthesis of thymidine triphosphate (dTTP) leading to deoxy nucleotide triphosphate (dNTP) pool imbalance, and results in accumulation of deoxyuridine triphosphate (dUTP) and fluorodeoxy uridine triphosphate (FdUTP), which lead to genomic DNA replication defects including DNA mismatch and altered replication fork progression eliciting DNA damage cell responses and, ultimately, cell death^5–8^.

Translation of these pharmacologic principles to the clinics led to regimens of FUra combined with high dose FA possessing greater antitumor efficacy than single FUra that are used for treatment of patients with colorectal, gastric, and pancreas adenocarcinomas^9,10^. However, further attempts at improvement of the anticancer effect of the modulation did not convincingly succeed. Probably, the effect of the combination has reached a limit that could not be overcome by using the pure levorotatory [6S]-stereoisomer of folinic acid instead of the [6R,S] mixture of stereoisomers^11^, by increasing the dose of folinic acid^11^ or through changes in schedule, and duration of administration of compounds^12^.

**Figure 1:**
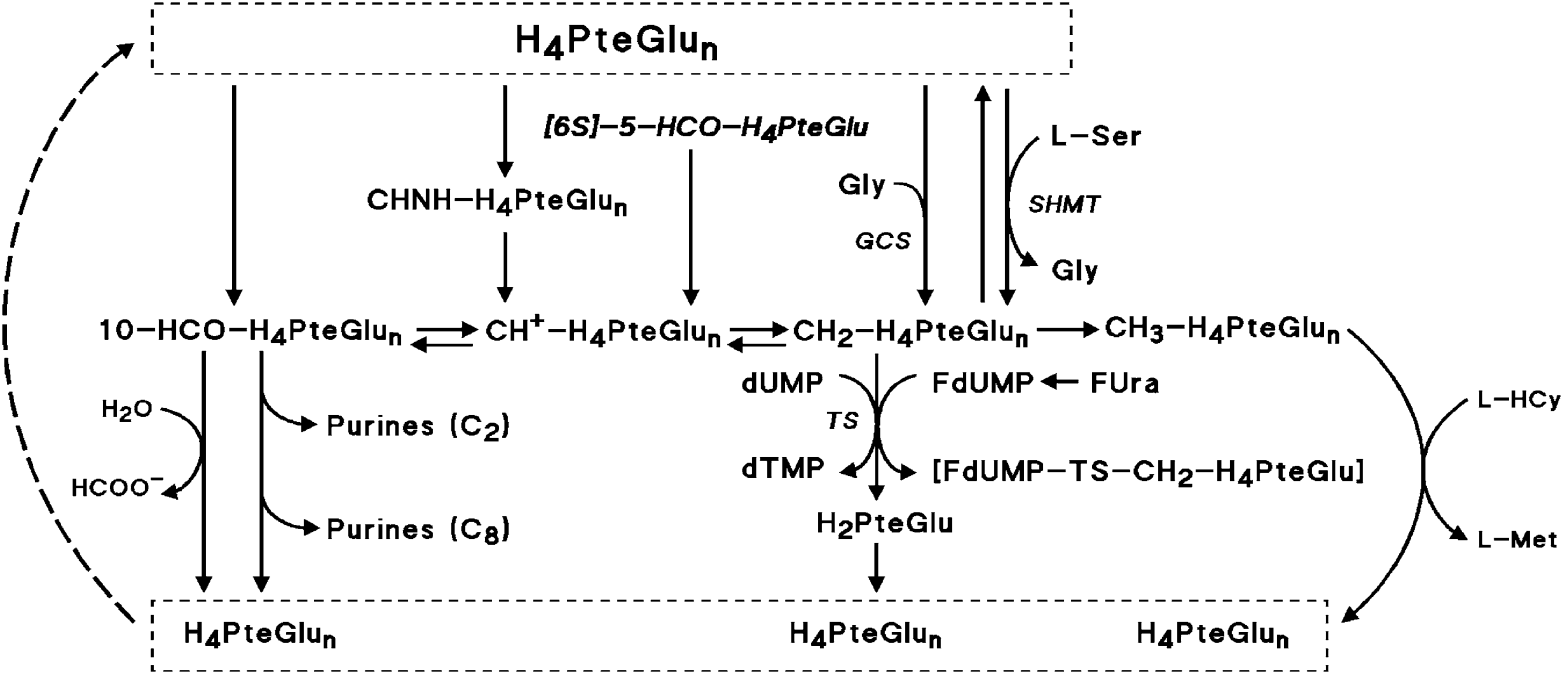
Selected pathways of folates, and FdUMP-mediated thymidylate synthase inhibition. *Folates* H_2_PteGlu: 7,8-di hydro pteroylglutamate; H_4_PteGlu: 5,6,7,8-tetra hydro pteroylglutamate; CH_2_-H_4_PteGlu: 5,10-methylene tetra hydro pteroylglutamate; CH_3_-H_4_PteGlu: 5-methyl tetra hydro pteroylglutamate; CH^+^-H_4_PteGlu: 5,10-methenyl tetra hydro pteroylglutamate; 10-HCO-H_4_PteGlu: 10-formyl tetra hydro pteroylglutamate; CHNH-H_4_PteGlu: 5-formimino tetra hydro pteroylglutamate; [6S]-5-HCO-H_4_PteGlu: [6S]-5-formyl tetra hydro pteroylglutamate ([6S]-folinic acid). *Enzymes* TS, thymidylate synthase; SHMT, serine hydroxymethyltransferase (pyridoxal 5’-phosphate-dependent enzyme, including the cytoplasmic SHMT1 and the mitochondrial SHMT2 isoforms); GCS, glycine cleavage system (mitochondrion). *Other compounds and substances involved in TS inhibition* dUMP, deoxy uridine monophosphate; dTMP, thymidine monophosphate; L-Ser, L-serine; Gly, glycine; L-HCy, L-homocysteine; L-Met, L-methionine; HCOO^-^, formate; FUra, 5-fluorouracil; FdUMP, fluorodeoxyuridine monophosphate; [FdUMP-TS-CH_2_-H_4_PteGlu], the ternary complex resulting in inhibition of TS.

Enhancement of the cytostatic activity of the fluoropyrimidines by reduced folates varies among cancer cells. Differences were associated with capacities for folate polyglutamation^13^ and for expansion of CH_2_-H_4_PteGlu pools. Studies of CH_2_-H_4_PteGlu concentration changes in cells exposed to 5-HCO-H_4_PteGlu either in [6R,S]- or [6S]-form, to 5-methyl tetra hydro pteroylglutamate (CH_3_-H_4_PteGlu), or to 5,6,7,8-tetra hydro pteroylglutamate (H_4_PteGlu), have demonstrated that supplementation of cancer cells with any amount of these folates results in limited increase of CH_2_-H_4_PteGlu concentration up to levels far below that required to increase the tightness of FdUMP binding to TS for optimum stability of the ternary complex, followed by rapid decline after discontinuation of folate exposure^2,3,13–22^. Poor intracellular expansion of CH_2_-H_4_PteGlu pools results from the rapid turnover of folates in cancer cells^23^ including the irreversible reduction of CH_2_-H_4_PteGlu to CH_3_-H_4_PteGlu and may be a consequence of limited production of CH_2_-H_4_PteGlu from H_4_PteGlu (Fig. 1). Synthesis of CH_2_-H_4_PteGlu from H_4_PteGlu results from two pathways. One is the transfer of Cβ of serine to H_4_PteGlu catalyzed by serine hydroxymethyl transferase (SHMT), a ubiquitous pyridoxal 5’-phosphate (PLP)-dependent enzyme that is the major source of one-carbon units for cellular metabolism^24–27^. The second pathway is the Glycine Cleavage System that catalyzes glycine cleavage up to formation of CH_2_-H_4_PteGlu in mitochondria^28,29^. The biochemical rationale for augmenting the cytotoxicity of the fluoropyrimidines by reduced folates and vitamin B6 in tandem lies in the low affinity for binding of apo-SHMT to PLP. SHMT from various mammalian sources including man binds to cofactor with K_d_ from 250 nM to as high as 27 µM^24–27^ while naturally occurring PLP levels in erythrocytes vary approximately from 30 to 100 nmol/L of cells^29,30^, which indicates that SHMT activity should be sensitive to intracellular PLP concentration changes. Folate-mediated one-carbon metabolism modifications and changes in SHMT activity related to vitamin B6 availability were described in rats fed with vitamin B6 deficient diet. Under this condition, animals had reduced PLP levels and SHMT activity in liver accompanied by decreased methylation of homocysteine to methionine with methyl groups from serine i.e., resulting from decreased SHMT-catalyzed synthesis of CH_2_-H_4_PteGlu, and subsequently of CH_3_-H_4_PteGlu, a cofactor of the cobalamin-dependent methionine synthase (Fig. 1)^31^. Rats fed with graded amounts of pyridoxine (PN) had liver cytosolic and mitochondrial SHMT activities increased with increasing dietary PN concentration^32^. Addition of exogenous PLP in vitro to measure the fraction of enzyme in apo form, markedly augmented SHMT activity in liver cell extracts of animals fed with all levels of dietary PN studied^32^. Similarly, the human MCF-7 mammary carcinoma cells cultured in PN deficient medium exhibited decrease in PLP levels, SHMT activity, and S-adenosyl methionine levels compared to that found in cells grown in standard cell culture medium^26^. Inclusion of exogenous PLP in cell extracts greatly increased SHMT activity which indicates, as from data in rat liver described above^32^, that a large proportion of SHMT pools lie as inactive apoenzyme^26,32^.

From these data we hypothesized that, in tumors, naturally occurring PLP levels are too small to allow intracellular SHMT-dependent conversion of H_4_PteGlu into CH_2_-H_4_PteGlu in amounts required to improve inhibition of TS by FdUMP by increasing stability of the ternary complex^33^. To test for variations of SHMT activity resulting from PLP level changes in cancer cells, we conducted experiments in the human colon carcinoma HT29, and HCT116 cell lines, and in the murine leukemia L1210 cell line in vitro to investigate for interactions between FUra, FA, and PLP on cell growth^33^. Supplementation of cancer cells exposed to FUra with high concentrations of PLP and FA in tandem strongly potentiated the cytotoxic activity of FUra in the three cell lines and resulted in powerful growth inhibiting synergistic interaction in HT29 and in L1210 cells, while summation was found in HCT116 cells. These findings support the hypothesis of expansion of CH_2_-H_4_PteGlu pools resulting from increase in SHMT activity by supplying cancer cells with PLP.

**Figure 2:**
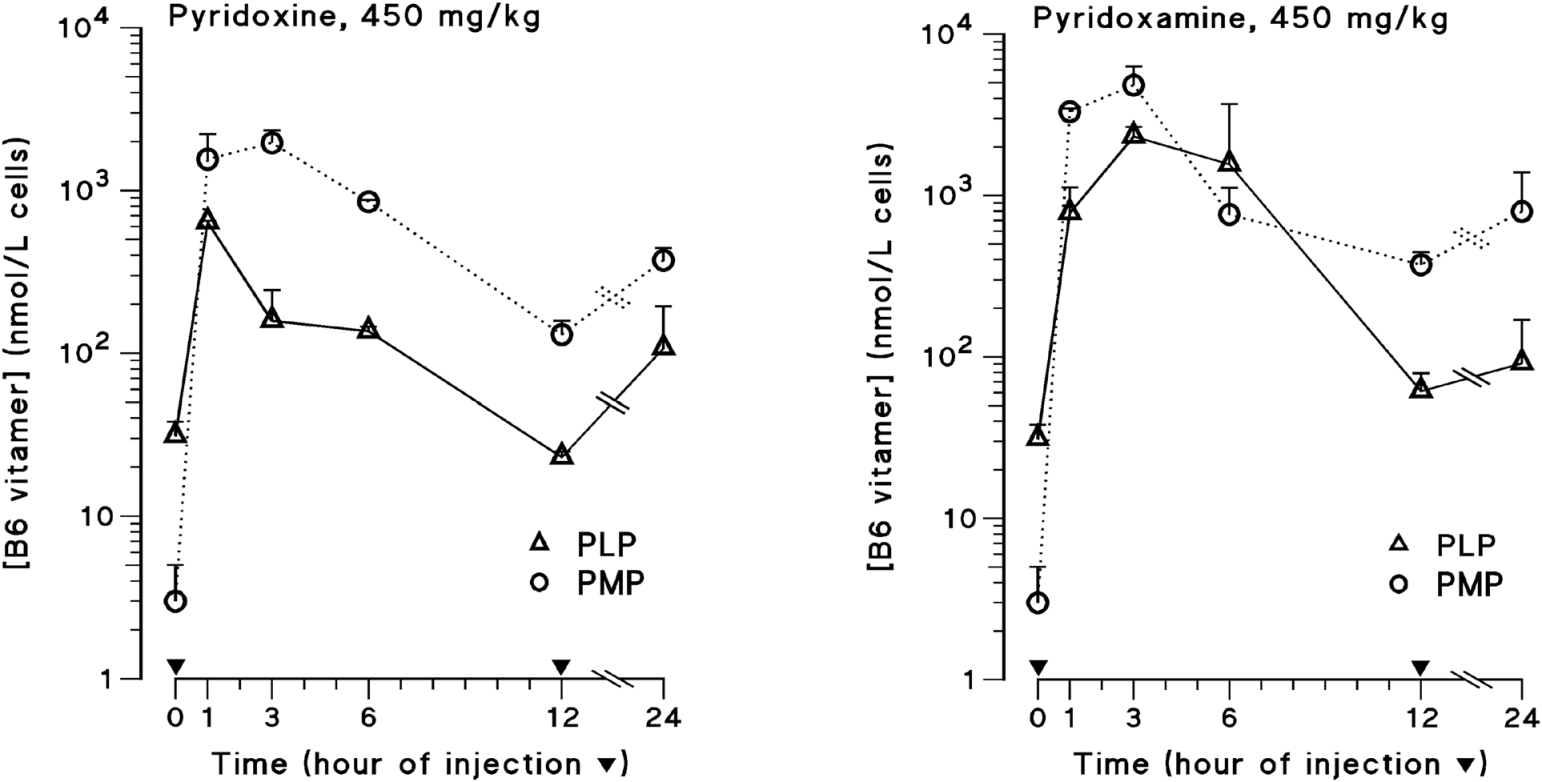
Erythrocyte pharmacokinetics of B6 vitamers after parenteral administration of high-dose pyridoxine or pyridoxamine in mice. Mouse erythrocyte levels of pyridoxamine 5’-phosphate (PMP; open circles), and pyridoxal 5’-phosphate (PLP; open triangles) were measured after intraperitoneal high-dose pyridoxine (PN) or pyridoxamine (PM). BALB/c mice were given PN or PM at 450 mg/kg at time 0 only, or twice at time 0 and after 12 h from start. For each unphosphorylated B6 vitamer explored, measurements of PMP and PLP were done after 1, 3, 6, 12, and 24 h from start of the experiment. Vertical bars indicate S.E. Table below indicates erythrocyte PLP peak concentration levels and PLP area under the concentration *vs* time curve in 12 h from injection.

Intracellular pharmacokinetics experiments were conducted in mice to study the physiologic capacities for the biochemical modulation of FUra by vitamin B6 to be achieved in vivo by expanding intracellular PLP pools, and for possible limitations^33^. BALB/c mice were given high doses of pyridoxamine (PM) or pyridoxine (PN) at 450 mg/kg by intraperitoneal route at time 0 only (*t*0), or twice at time 0 and after 12 h from start (*i.e.*, at times *t*0 and *t*12h) before being sampled at regular intervals. Studies determined that erythrocyte levels of PLP after parenteral administration of each unphosphorylated B6 vitamer rose to concentration levels within the range of K_d_ values of SHMT binding to cofactor^24–27^, and that newly synthesized PLP was rapidly cleared from cells^33^. Levels decreased to reach baseline concentrations by 12 h after injection, with no measurable cumulative effect when administration of B6 vitamer was repeated at 12-h interval. Rapid decline of intracellular PLP levels after vitamin B6 administration was also reported in man^29,30^. From these data, we thought that administration of high-dose unphosphorylated B6 vitamer to patients treated with FUra and FA would increase intracellular PLP levels within tumors, leading to augmentation of CH_2_-H_4_PteGlu synthesis resulting in long-term TS inhibition and enhanced antitumor effect. Additional analysis of data obtained from these experiments^33^ determined that intraerythrocytic PLP peak concentration levels, and PLP area under the concentration *vs* time curve in 12 h from injection (AUC_t0-12 h_ in nmol/L cells**∙**12 h) in mice that received intraperitoneal PM were 3.7- and 6.7-fold greater than that measured in animals having received PN, respectively (Fig. 2). An explanation for the significant discrepancy of intracellular pharmacokinetics between these unphosphorylated B6 vitamers could lie in differences for conversion in cofactor. Both PM and PN are phosphorylated by the ATP-dependent pyridoxal kinase (PLK) in PMP and PNP respectively, and then oxidized to PLP by the flavin mononucleotide (FMN)-dependent pyridoxine (pyridoxamine) 5′-phosphate oxidase (PNPOx)^34–37^, whose affinity for PMP was reported greater than that for PNP; measured K_m_ of PNPOx for PMP, and for PNP were 1.0 µM, and 1.8 µM, respectively^37^. In addition to this catalytic sequence that is common to both vitamers, PMP was described to be reversibly converted in PLP through a pyridoxamine aminotransferase-catalyzed pathway^34–36^. Present findings suggest that parenteral PM may possess an advantage over PN to increase intracellular PLP pools.

**Table:** 

Intracellular pharmacokinetics parameter	PN, 450 mg/kg i.p.		PM, 450 mg/kg i.p.	
	PMP	PLP	PMP	PLP
Mean peak B6 vitamer concentration (nmol/L cells)	1961	637	4829	2326
B6 vitamer AUC_t0-12h_ (nmol/L cells 12h)	11,597	2231	19,356	14,988

Chart 2 of the Figure is republished with permission of the American Society for Pharmacology and Experimental Therapeutics (ASPET), from Enhancement of 5-Fluorouracil Cytotoxicity by Pyridoxal 5'-Phosphate and Folinic Acid in Tandem by Machover, D. et al., Journal of Pharmacology and Experimental Therapeutics, August 2018, 366 (2) 238–243^33^; permission conveyed through Copyright Clearance Center, Inc.

Chemotherapy regimens for treatment of patients with breast carcinoma, mostly combining two to four cytostatics, often administered in sequence, include anthracyclines, taxanes, vinca alkaloids, alkylating agents, platinum coordination compounds, and fluoropyrimidines. Currently used standard regimens produce effective but limited antitumor effect in patients with tumors in advanced stage whose response rates range frequently between 40 and 60%, with approximately10 to 15% of patients attaining a complete response^38,39^. Phase II studies of FUra and folinic acid administered either as single agents or in combination with one or two cytostatics for patients with advanced breast carcinoma led to favorable results^40–49^. In patients who had not received prior chemotherapy for treatment of advanced breast carcinoma, mean response rate was 39% (range, 36–41%) in patients treated with FUra and folinic acid as single agents; 64% (range, 59–70%) in patients who received FUra, and FA combined with vinorelbine; 72% (range, 51–92%) in patients who received FUra, and FA combined with paclitaxel; and 64% (range, 35–83%) in patients treated with FUra, and FA in combination with an anthracycline plus cyclophosphamide. Mean overall complete response rate reported in these studies was 12% (range, 2–27%). In patients with prior chemotherapy for advanced breast carcinoma who received FUra and FA as single agents or in combination, response rates ranged from 29 to 51%. However, up to now FUra plus FA modulation-based schemas have not been widely recognized as components of current treatment options for breast carcinoma patients.

We report herein a translational pilot study in patients with breast carcinoma in advanced stages who were not amenable to resection or radiotherapy with curative intent, and whose standard treatment regimens included a combination of FUra and FA, consisting in addition of pyridoxine (PN) in high dose to these combination regimens. The present pilot study is the first step before planning clinical trials.

Pyridoxine in high doses used for treatment of various conditions in man was reported to be safe**,** although it caused sensory peripheral neuropathy when it was administered in extremely high doses for long periods of time^50,51^. From these prior data, we thought that vitamin B6 administered in short time courses followed by drug-free intervals in doses far below that reported to be toxic in man, was not likely to expose patients to increased risk of neuropathy. However, during the study we proceeded with progressive dose escalation of pyridoxine and were particularly cautious on neurologic signs and symptoms.

Modulation of FUra by high-dose FA and PN in tandem was used in a pilot study for treatment of patients with unresectable or metastatic colorectal adenocarcinoma, pancreas adenocarcinoma, and squamous cell carcinoma of the esophagus^52^. Addition of high-dose PN to standard treatment regimens comprising FUra and FA led to high rate of antitumor responses of early onset and great magnitude with no detrimental effect on toxicity from that expected using these regimens in absence of PN.

## Materials and methods

The study was approved by the Medical Oncology Department board in Paul-Brousse Hospital, Assistance Publique-Hôpitaux de Paris, and University Paris-Saclay. It was conducted in accordance with the basic principles of the Declaration of Helsinki. All the patients were informed of the rationale, potential benefits, and risks of the treatment. Written informed consent to study participation was obtained from all patients. Study included 27 patients with breast adenocarcinoma in advanced stages that were entered in a single clinical center from December 2014 to February 2021. Patients presented either with highly advanced tumor of the breast together with ipsilateral lymph node involvement of variable extent, accompanied or not with bone, nodal and/or visceral metastases, or with metastases only.

### Patients

Patients with ductal or lobular adenocarcinoma of the breast in advanced stages carrying poor prognostic features who did not receive prior chemotherapy as well as those who had previously received no more than two prior lines of chemotherapy were included in the study. Previously treated patients could have received one chemotherapy regimen as pre-operative (neo adjuvant) or post-operative adjuvant treatment, and/or one line of treatment for advanced disease. Prior chemotherapy had to be terminated at least 3 months before entering the study. Previous hormone therapy of any type was admitted. Owing to the great extent of tumor at presentation and poor performance status in many, patients could not be eligible for surgery or radiotherapy with eradication intent, nor for any available investigational therapy (Table 1; Tables 1 and 2 Supplementary).

**Table 1:** Characteristics of patients with advanced breast carcinoma treated with regimens including 5-fluorouracil, folinic acid, and pyridoxine in tandem.

Category^a^	Patient	Age	AJCC Staging of primary (T) and ipsilateral nodes (N) [Characteristics of primary]^b,c^	Estimated extent of metastases (site, and approximate No of metastases by site)^d^	AJCC stage	ECOG PS
I	1	47	T4d, N3c [LC EEII Ki67:10 ER+ PR+]	Nodes (neck, 1); bone (multiple); pectoral muscle	IV	0–1
	2	50	T3, N2 [DC EEII Ki67:30 ER+ PR+]	Bone (multiple)	IV	0–1
	3	63	T4d, N1 [DC EEIII Ki67:70 ER+ PR−]	Absence	IIIB	0–1
	4	49	T3, N3c [DC EEII Ki67:40 ER+ PR+]	Nodes (mediastinum, 11; axilla, 2)	IV	2
	5	53	T2, N3c [LC ER+ PR+]	Nodes (mediastinum, 4; abdomen, 5); bone (disseminated); peritoneum; pleura; spleen	IV	4
	6	37	T3, N2 [DC EEIII Ki67:35 ER+ PR+]	Bone (1)	IV	2
	7	44	T4c, N2 [DC EEII Ki67:22 ER+ PR+]	Nodes (mediastinum, massive); lung (5); pleura, pericardium (massive); skin (nodules; ulcer) ; bone (multiple)	IV	3
	8	48	T4d, N3 [DC EEII Ki67:30 ER+ PR+]	Nodes (mediastinum, 3); pectoral muscle	IV	3
	9^b^	62	N2a [DC EEIII Ki67:35 ER+ PR−]^b^	Skin (nodules); pectoral muscle	IV	0–1
	10	41	T4d, N3a [DC EEII Ki67:40 ER+ PR−]	Bone (1)	IV	2
	11^b^	43	N0 [DC EEIII Ki67:10 ER+ PR−]^b^	Skin (nodules, > 4)	IV	0–1
	12	43	T4d, N3c [DC EEIII Ki67:60 ER+ PR−]	Nodes (mediastinum, 6); liver (1); pleura; axillary muscles	IV	0–1
II	13	52	T4d, N3b [DC EEII Ki67:25 ER+ PR−]	Nodes (mediastinum, 1)	IV	0–1
	14	47	T4d, N2 [DC EEIII Ki67:30 ER− PR−]	Absence	IIIB	0–1
	15	62	T4d, N3b [DC EEIII Ki67:70 ER− PR−]	Absence	IIIC	3
	16	55	T2, N2 [DC EEII Ki67:40 ER+ PR−]	Liver (innumerable); lung (innumerable); bone (disseminated)	IV	4
	17	51	T4d, N3c [DC EEI Ki67:80 ER- PR-]	Nodes (neck, 4; axilla, 1); thorax wall (massive)	IV	3
	18	68	T2, N3c [DC Ki67:20 ER+ PR−]	Nodes (mediastinum, 7; axilla, 1); liver (innumerable); bone (disseminated); cranial nerve involvement	IV	4
III^b^	19	75	[DC ER+ PR−]	Bone (multiple); cranial and peripheral nerve involvement	IV	3
	20	47	[LC EEII ER+ PR−]	Nodes (mediastinum, > 3; abdomen, 2); liver (innumerable)	IV	2
	21	68	[DC EEII ER+ PR−]	Nodes (mediastinum, 1)	IV	0–1
	22	45	[DC EEII Ki67:10 ER+ PR−]	Nodes (mediastinum, 4); liver (3); lung (1); bone (multiple)	IV	0–1
	23	40	[DC EEII Ki67:90 ER− PR−]	Nodes (mediastinum, 2; supraclavicular, 1); liver (2); bone (multiple); thorax wall	IV	0–1
	24	42	[DC EEII Ki67:25 ER+ PR+]	Lung (innumerable); bone (disseminated)	IV	3
	25	54	[DC EEIII Ki67:80 ER− PR−]	Nodes (neck, 3; axilla, 1); liver (1); epidural spinal cord compression	IV	4
	26	56	[DC EEII Ki67:70 ER+ PR−]	Nodes (mediastinum, 3); liver (1); lung (7)	IV	0–1
	27	54	[DC EEIII Ki67:60 ER− PR−]	Nodes (axilla, 2); skin (nodules)	IV	2

^a^Categories of patients are I, previously untreated patients whose tumors did not overexpress HER2 (1–12); II, previously untreated patients whose tumors overexpressed (3+) HER2 (13–18); and III, patients who had received prior chemotherapy whose tumors did not overexpress HER2 (19–27).

^b^Patient with prior mastectomy.

^c^*LC* lobular carcinoma, *DC* ductal carcinoma, *EE* Elston-Ellis pathologic grade, *Ki67* expression in percent of cancer cells, *ER* estrogen receptors, *PR* progesterone receptors.

^d^Axilla refers to metastatic lymph nodes in axilla contralateral to primary.

Twenty-seven patients aged 37–75 years old (median, 50 years) were included in the study (Table 1). Of 18 patients who had not received prior chemotherapy, 6 had tumors that overexpressed (3+) the Human Epidermal Growth Factor Receptor-2 (Her2/neu; HER2) as assessed by immunohistochemistry and 12 had tumors that did not. Of these 18 patients, 9 presented with locally advanced unresectable tumor accompanied with bone, nodal, soft tissue, and/or visceral metastases, and 9 had inflammatory carcinoma of who 6 had distant metastases as well; AJCC anatomic stages were IIIB, IIIC, and IV in 2, 1, and 15 patients, respectively. Two of the 12 patients with tumors that did not overexpress HER2 who had not received prior chemotherapy presented with skin permeation nodules in one patients and skin permeation nodules, muscle invasion and ipsilateral axillary lymphadenopathy in the other, developed in the anatomical area of prior exclusive mastectomy performed 1 and 21 years before relapse (Table 1; Tables 1 and 2 Supplementary). Of the 18 previously untreated patients, 15 had tumors that expressed ERs, and 3 had tumors that did not (Table 1). Nine patients with stage IV breast carcinoma diagnosed 1.5 to 25 years (mean, 8.4 years) before entering the present study had received prior chemotherapy. Of these, 4 have had prior neo adjuvant or adjuvant chemotherapy only, 3 had first-line chemotherapy for advanced disease only, and two patients had both, neo adjuvant or adjuvant chemotherapy with subsequent first-line chemotherapy for treatment of metastatic disease. All nine previously treated patients have had prior anthracycline-containing chemotherapy and 8 had taxanes as well. Eight of these 9 patients had also received FUra as part of their previous regimens of chemotherapy, including 4 who had FUra combined with folinic acid. None of them had tumors with HER2 overexpression. Of the 9 patients, 6 had tumors that expressed ERs, and 3 had said triple negative carcinoma. In addition to prior chemotherapy, the six previously treated patients whose tumors expressed ERs had previous endocrine therapy in various forms. Eight of 9 patients who had received prior chemotherapy presented with measurable tumor consisting in nodal, bone, soft tissue and/or visceral metastases, including one patient who had also locally advanced disease. One patient had bone metastases only (Table 1; Tables 1 and 2 Supplementary). All patients had prior total or partial mastectomy (Table 1).

High initial plasma tumor marker levels (≥ twice the upper limit value) were found in 15 patients who had elevated CA15-3, together with high CEA, and/or CA125 levels in 7 patients, and 8 patients, respectively. Great tumor burden was recorded in most patients (Tables 1, 2; Table 1 and 2 Supplementary). Eastern Cooperative Oncology Group (ECOG) performance status (PS) scores at presentation were 0–1, 2, and 3–4 in 12, 5, and 10 patients, respectively (Table 1). Two patients carried deleterious germline BRCA2 gene mutations.

**Table 2:** Results of therapy in patients with advanced breast carcinoma treated with regimens comprising 5-fluorouracil, folinic acid, and pyridoxine in tandem.

Category^a^	Patient	Regimens^b^ comprising FUra, FA and PN in tandem given in succession from A to C [No. of courses of each regimen]			Median dose of PN^c^	Time to response^d^ (Mo.)	Antitumor activity			CA15-3 start/after treatment (U/ml)	PFS^i^ (Mo.)
		A	B	C			RECIST^e^	PERCIST^f^	Pathologic (AJCC)^g^		
I	1	AVCF [6]	TCbF [6]	VCbF [10]	3	4.0	− 100	− 100	–	166/24	22+
	2	FAC [4]	TCbF [4]	VCbF [5]	1	4.5	− 100	− 100	–	–	45+
	3	AVCF [4]	TCbF [5]	VCbF [3]	1	4.6	− 100	− 100	ypT0N0	–	44+
	4	FAC [6]	TCbF [6]	VCbF [6]	3	2.3	− 98	− 100	–	272/29	21+
	5	VCbF [3]	TCbF [6]	VCbF [21]	2	4.7	− 98	− 100	–	2212/123	22
	6	AVCF [6]	TCbF [1]	VCbF [8]	3	3.3	− 96	− 100	ypT1bN0	–	17+
	7	AVCF [6]	TCbF [5]	VCbF [1+]	3	2.4	− 93	− 94	–	1017/31	9+
	8	AVCF [6]	TCbF [7]	VCbF [6]	2	4.4	− 89	− 87	–	849/24	51
	9^h^	TCbF [5]	VCbF [6]		2.5	3.4	− 89	− 84	ypN1aM0^h^	–	33+
	10	AVCF [5]	TCbF [9]		1	5.3	− 79	− 85	ypT1cN1a	–	38
	11^h^	TCbF [8]	VCbF [6]		1	3.9	− 64	− 50	ypN0M1^h^	–	55+
	12	TCbF [7]			1	2.5	− 62	Na	–	782/73	7
II	13	TCbF [8]	VCbF [3]		1	3.9	− 100	− 100	ypT0N0	62/25	44+
	14	TCbF [8]	VCbF [4]		2	1.7	− 100	− 100	ypT0N0	–	71+
	15	TCbF [8]	VCbF [4]		2	4.2	− 100	− 93	ypT0N0	–	67+
	16	TCbF [9]	VCbF [15]		3	2.4	− 100	− 100	ypT1bN0	14,750/29	27
	17	TCbF [12]	VCbF [39]		1	8.4	− 98	− 100	–	–	70+
	18	TCbF [8]	VCbF [21]		3	2.0	− 98	Na	–	422/26	23+
III^h^	19	TCbF [9]			2	2.1	Na	− 100	–	152/79	54
	20	TCbF [8]			1	2.8	− 100	− 100	–	1272/12	13
	21	VCbF [18]			2	2.4	− 100	− 93	–	116/29	34
	22	TCbF [12]			3	6.8	− 94	− 47	–	126/46	12
	23	VCbF [12]			1	2.6	− 91	− 100	–	72/13	28+
	24	TCbF [18]			2	5.4	− 88	− 100	–	101/14	30^i^
	25	TCbF [23]			1	3.1	− 88	− 100	–	–	15
	26	TCbF [21]			2	3.3	− 81	− 91	–	–	27
	27	TCbF [8]			1	–	45	Na	–	–	–

^a^Categories comprise I, previously untreated patients whose tumors did not overexpress HER2 (1–12); II, previously untreated patients whose tumors overexpressed (3+) HER2 (13–18); and III, previously treated patients whose tumors did not overexpress HER2 (19–27).

^b^Composition of regimens is described in text.

^c^Median dose of PN preceding each injection of FUra and FA (× 10^3^ mg/day).

^d^Time to attain antitumor response, i.e., reduction in sum of diameters by ≥ 30%.

^e^Patients who attained a PR accompanied by disappearance of most metastases had RECIST (Response Evaluation Criteria in Solid Tumors) values calculated by size comparison of persisting tumors at the time of assessment with these same tumors before treatment.

^f^Percent variation in peak standard ^18^FDG uptake value normalized by lean body mass (SUL_peak_) assessed by PET scan (PERCIST; Positron Emission Tomography (PET) Response Evaluation Criteria in Solid Tumors).

^g^Pathologic response was assessed by mastectomy or by locoregional resection with eradication intent in patients with prior mastectomy.

^h^Patient with prior mastectomy.

^i^EFS time in Patient 24. *Na* not assessed.

### Treatment

Patients received induction regimens of chemotherapy comprising a combination of FUra and FA employed in our standard practice that were indicated for treatment of their disease and specific clinical condition, supplemented with pyridoxine in high doses accompanying each administration of FUra plus FA (Table 2). Four different combination regimens were used either alone or in sequence as indicated according to each category and/or clinical specificities of patients. Regimens were (a) *AVCF*, consisting in 4-day courses every 21 days of doxorubicin, 40 mg/m^2^ iv Day 1; vinorelbine, 25 mg/m^2^ iv Day 1; cyclophosphamide, 250 mg/m^2^/day iv Days 1–4; FUra, 400 mg/m^2^/day iv in 2 h, Days 1–4; and folinic acid (FA; [6R,S]-5-formyl tetra hydro pteroylglutamate; [6R,S]-5-HCO-H_4_PteGlu), 200 mg/m^2^/day iv in 15’ Days 1–4; (b) *FAC,* consisting in 1-day courses every 21 days of doxorubicin, 40 mg/m^2^ iv Day 1; cyclophosphamide, 500 mg/m^2^ iv Day 1; FUra, 500 mg/m^2^ iv in 2 h; and FA, 200 mg/m^2^ iv in 15’ Day 1; (c) *TCbF*, consisting of 4-day courses every 21 days of paclitaxel, 175 mg/m^2^ iv Day 1; carboplatin, AUC = 5 mg/ml min iv Day 1; FUra, 400 mg/m^2^/day iv in 2 h, Days 1–4; and FA, 200 mg/m^2^/day iv in 15’ Days 1–4; and (d) *VCbF* consisting of 4-day courses every 21 days of vinorelbine, 25 mg/m^2^ iv Day 1; carboplatin, AUC = 5 mg/ml min iv Day 1; FUra, 400 mg/m^2^/day iv in 2 h, Days 1–4; and FA, 200 mg/m^2^/day iv in 15’ Days 1–4. All treatment courses were accompanied by granulocyte colony-stimulating factor (G-CSF) beginning the first day of each interval between courses.

Doxorubicin comprised in *AVCF* and *FAC* regimens was suspended in case of ≥ 10% decrease in left ventricular ejection fraction from baseline value. Paclitaxel included in the *TCbF* regimen was suspended when symptoms of sensory peripheral neuropathy (SPN) consisting in permanent hypoesthesia, paresthesia and/or dysesthesia of any intensity, and/or limb pain were first recorded. Vinorelbine-containing chemotherapy (i.e., *VCbF*) was indicated for patients who had severe hematopoietic impairment or prior taxane-induced toxicity and was used in substitution for *TCbF* in cases of paclitaxel-induced SPN occurring in patients during the study.

Of twelve patients who were not previously treated and whose tumors did not overexpress HER2, 8 received an initial sequence of anthracycline-containing chemotherapy (4–6 courses), and then a succession of *TCbF* courses followed by *VCbF* in substitution to *TCbF* when needed for the reasons above. Anthracycline-containing chemotherapy was *AVCF* in 6 patients and *FAC* in 2. *AVCF* and *FAC* were avoided in 4 patients owing to hematologic impairment in 2 (one patient had myeloproliferative disorder and the other had profound myeloid cytopenia due to extensive bone marrow metastases and peripheral thrombocytopenia), and to mild cardiorespiratory dysfunction in two patients; these 4 patients received taxane- and vinorelbine-containing regimens only (Table 2). The six patients who had not been previously treated, and whose tumors overexpressed HER2 (3+) received a succession of *TCbF* courses combined with the anti HER2/neu humanized monoclonal antibodies trastuzumab (6 mg/kg iv every 21 days), and pertuzumab (420 mg/patient iv every 21 days). Induction chemotherapy for the nine patients who had been previously treated consisted in *TCbF* in seven patients and *VCbF* in two (Table 2). Premenopausal patients, whose tumors expressed ERs, received long-term luteinizing hormone-releasing hormone analog (LHRHa).

Vitamin B6 is the generic name that encompasses six interconvertible compounds (i.e., B6 vitamers), namely pyridoxine (PN); pyridoxamine (PM); pyridoxal (PL); and their respective 5’ phosphorylated forms, pyridoxine 5’-phosphate (PNP), pyridoxamine 5’-phosphate (PMP), and the cofactor pyridoxal 5’-phosphate (PLP)^29,35,36^. Pyridoxine hydrochloride, the only available marketed parenteral B6 vitamer for clinical use (in 250 mg vials) was injected iv in 30’ preceding each injection of FA and FUra for a number of days defined by the schedule of the regimen used (i.e., 4 consecutive days in *AVCF*, *TCbF*, and *VCbF* regimens, and single day in the *FAC* regimen). Based on the pharmacokinetics data obtained in mice^33^, using approximate factors for converting doses in man from mouse data^53^, the daily dose of pyridoxine was augmented in patients over the duration of the present study from 1000 mg/day to a maximum of 3000 mg/day. The latter corresponds approximately to the high dose of PN of 450 mg/kg explored in mice as described above^33^; in these animals, it resulted in rise of intracellular concentrations of PLP to peak levels within the range of reported K_d_ values for binding of PLP to apo SHMT, the requirement that supports the rationale underlying the present clinical study (Fig. 2)^24–27,33^. The first starting dose of PN accompanying each administration of FUra and FA was 1000 mg/day. Then, we practiced stepwise intra patient dose escalation of pyridoxine by increments of 500–1000 mg/day in subsequent courses. In absence of any form of toxicity seeming attributable to the PN recorded in prior patients, the starting daily dose of PN in next patients was increased to 2000 mg/day, and then to a maximum of 3000 mg/day (Table 2).

In all three categories of patients described above, courses of *TCbF* were not limited in number a priori, and were being substituted by *VCbF* in cases of paclitaxel induced SPN (Table 2). Courses were repeated until antitumor response of estimated maximum degree was attained in a personalized way according to patient’s condition, tolerance to treatment, phenotypic tumor specificities, and decisions from referring oncologists and clinical meetings. Nine patients who achieved either a complete or a partial response of great magnitude allowing resection of residual tumor, were subjected to mastectomy or to locoregional resection with eradication intent, after which a limited number of postoperative courses was administered and then chemotherapy was discontinued (Table 2 and Fig. 5). Once chemotherapy was terminated, patients whose tumors expressed ERs received long-term aromatase inhibitor therapy accompanied with LHRHa in premenopausal patients, and those whose tumor overexpressed HER2 received three-weekly trastuzumab during one supplementary year. Progression- and event-free survival data for each patient are indicated (Table 2 and Fig. 5). Owing to the variable post-induction therapies received by patients, we mainly focused on magnitude and characteristics of antitumor responses achieved with regimens including FUra, folinic acid and pyridoxine in tandem to assess for their antitumor potency.

## Results

Antitumor response was assessed by studying variation of the sum of diameters of anatomically measurable tumors according to RECIST (Response Evaluation Criteria in Solid Tumors)^54^, and that of peak standard ^18^FDG uptake value normalized by lean body mass (SUL_peak_) of targets according to PERCIST (Positron Emission Tomography (PET) Response Evaluation Criteria in Solid Tumors)^55^, together with periodic clinical examination including follow-up of non-measurable tumor involvement, and measurement of plasma tumor markers. Assessment of pathologic response (TNM AJCC staging) was obtained from nine patients who had attained either a complete response or a partial response of high magnitude who were subjected to mastectomy or to locoregional resection with eradication intent. Focal pathologic assessment was obtained in three patients by imaging-oriented percutaneous biopsy of previously involved sites with persisting abnormal images after treatment.

Of twenty-seven patients included, 26 responded to therapy and one had progressive disease. Induction treatment resulted in antitumor responses of early onset and great magnitude.

### Patients who had not received prior chemotherapy whose tumors did not overexpress HER2

**Figure 3:**
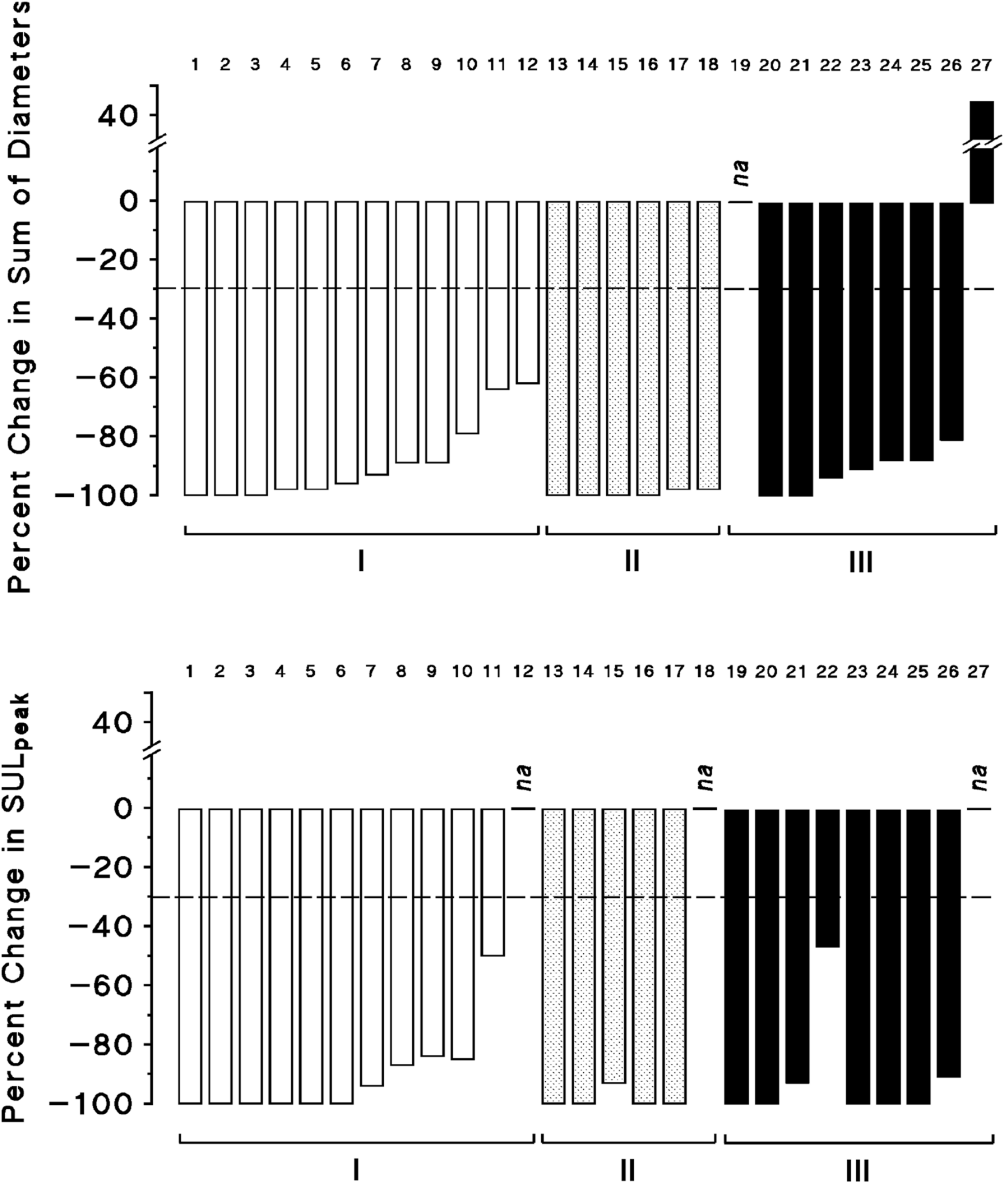
Magnitude of clinical and metabolic response in advanced breast carcinoma patients treated with regimens comprising FUra, FA, and pyridoxine in tandem. Patients in abscissa comprise I, previously untreated patients whose tumors did not overexpress HER2 (1–12); II, previously untreated patients whose tumors overexpressed (3+) HER2 (13–18); and III, previously treated patients whose tumors did not overexpress HER2 (19–27). In patients who had great numbers of targets who attained a partial response accompanied by disappearance of most metastases, calculations of percent reduction in sum of diameters (RECIST; Response Evaluation Criteria in Solid Tumors) were done by size comparison of remaining images at the time of assessment with these same tumor images present before treatment. Metabolic response was assessed by the percent variation in peak standard ^18^FDG uptake value normalized by lean body mass (SUL_peak_) obtained by PET scan (PERCIST; Positron Emission Tomography (PET) Response Evaluation Criteria in Solid Tumors). Three responders were not assessed by both methods (*na* in bar plots). The discontinuous line at − 30%, represents the limit between no change and antitumor response.

**Figure 4:**
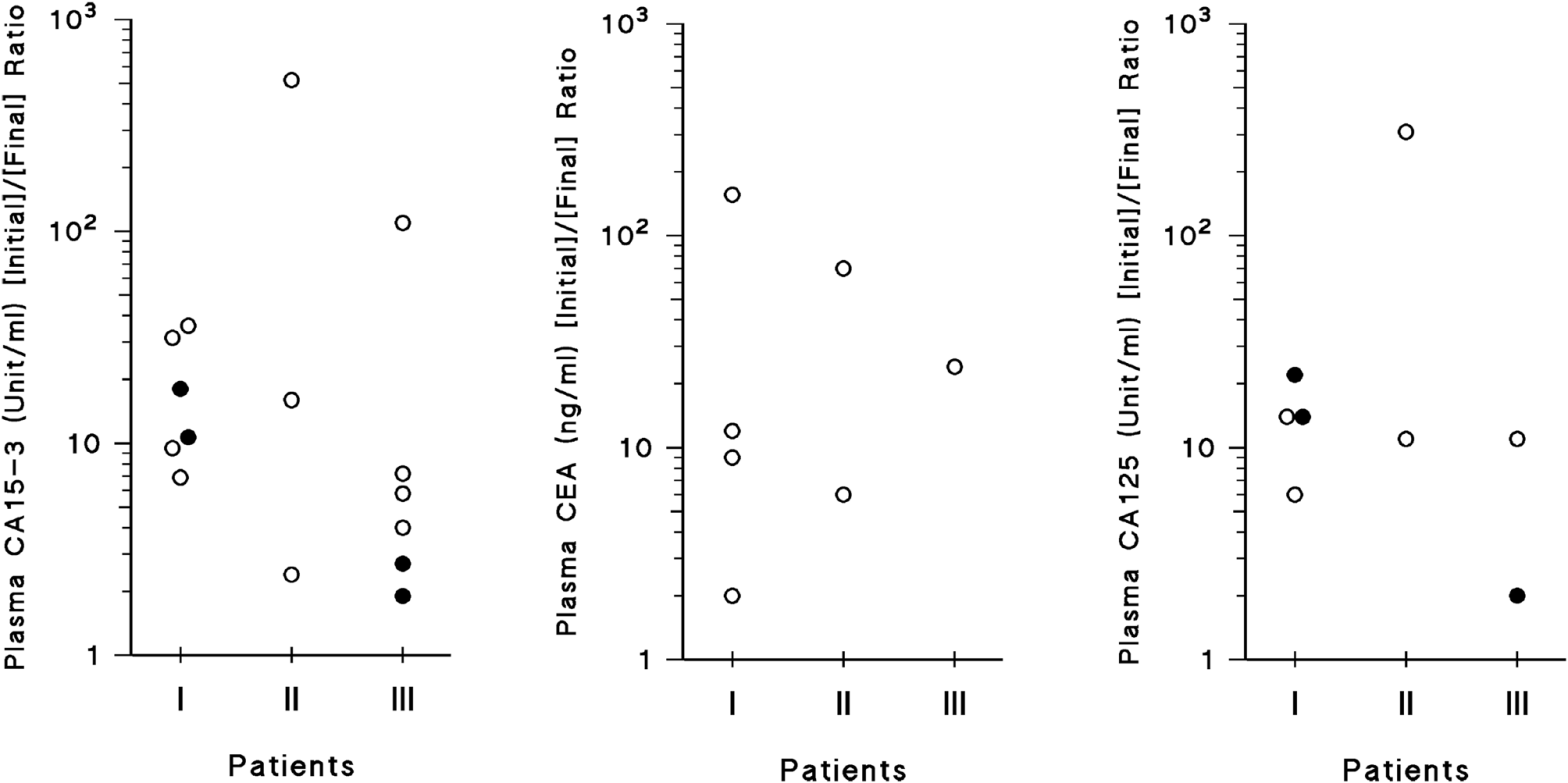
Variation of plasma tumor marker levels in patients with advanced breast carcinoma treated with regimens including FUra, high dose folinic acid and pyridoxine in tandem. Points in scattergram represent variation of plasma CA15-3, CEA, and CA125 levels under treatment as a ratio of the initial concentration to the final value at the time of antitumor activity evaluation. Only patients with plasma tumor markers whose initial levels were ≥ twice the upper limit of normal values are indicated. Open circles indicate patients whose markers attained levels equal or below the upper limits of normal values. Solid circles indicate patients whose marker levels decreased but remained above the upper limit of normal values. Groups of patients in abscissa comprise I, previously untreated patients whose tumors did not overexpress HER2; II, previously untreated patients with tumors overexpressing (3+) HER2; and III, previously treated patients whose tumors did not overexpress HER2.

**Figure 5:**
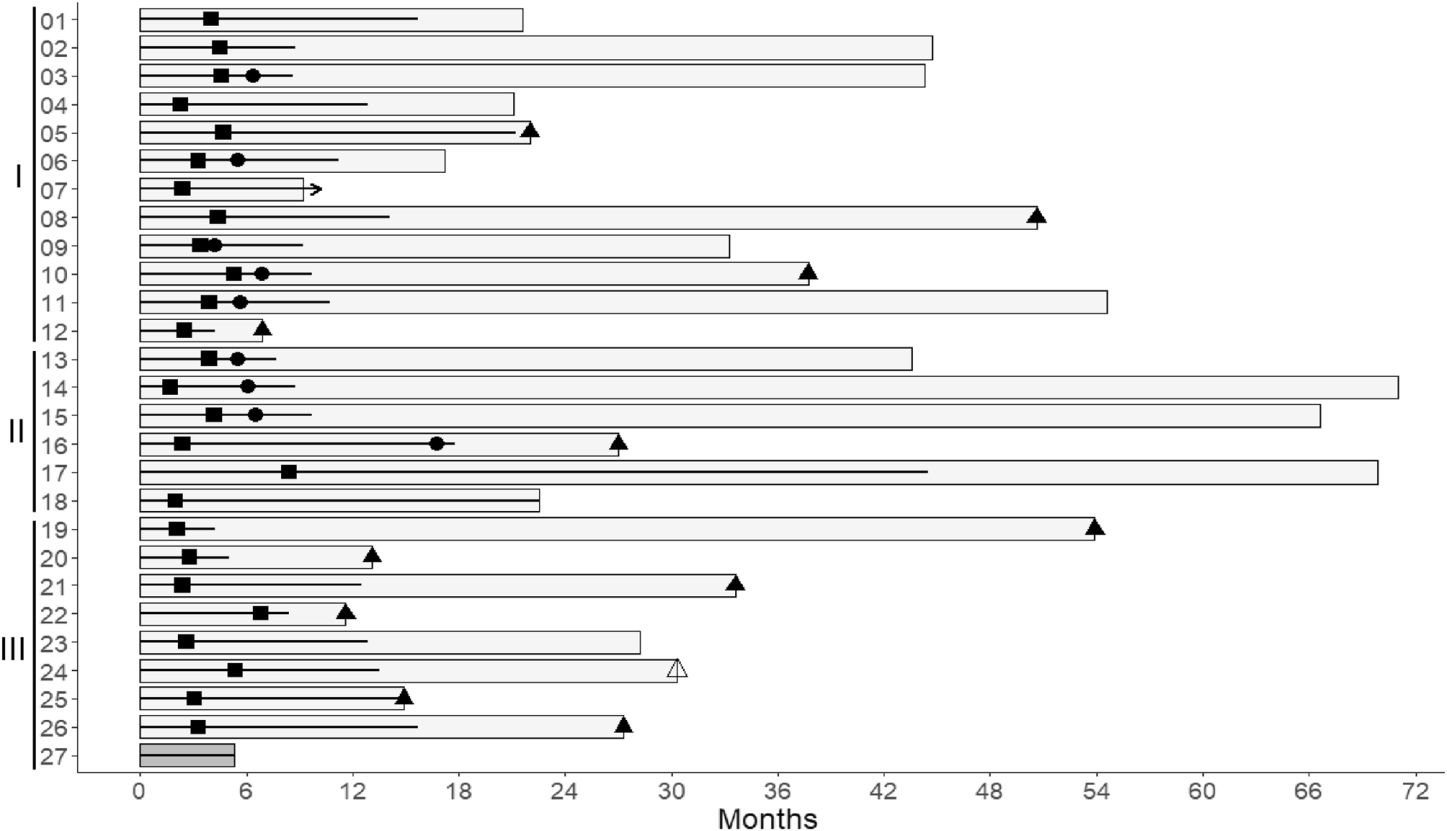
Chronological sequence of events in 27 patients with advanced breast carcinoma treated with regimens including FUra, folinic acid and pyridoxine in tandem. Groups of patients in ordinate comprise I, previously untreated patients whose tumors did not overexpress HER2 (1–12); II, previously untreated patients whose tumors overexpressed (3+) HER2 (13–18); and III, patients who had received prior chemotherapy whose tumors did not overexpress HER2 (19–27). Patients are numbered in the same order as in Tables 1, and 2, and in Fig. 3. Light grey bars represent progression-free survival (PFS) times in all patients except for Patient 24 where it represents event-free survival (EFS) time. Dark grey bar represents time required to final evaluation in the single patient that did not respond to therapy. Bold black lines within bars represent duration of treatment comprising FUra, folinic acid and pyridoxine in tandem, and arrow indicates ongoing treatment at the time of present evaluation. Solid squares indicate the time when a response to therapy was recorded, i.e., a reduction in sum of diameters by ≥ 30%. Solid circles represent the time when mastectomy or other type of surgery with eradication intent was performed. Solid triangles indicate the time when tumor progression was recorded in prior responders to therapy. Open triangle indicates time of event leading to withdrawal from study in a single patient with persisting response to therapy.

Twelve patients were included in this group. Ten patients were included at initial diagnosis and 2 patients were treated for relapse that occurred 1 year (Patient 11), and 21 years (Patient 9) after exclusive mastectomy (Table 1). Of the 12 patients, 3 attained clinical CRs and 9 had PRs with great tumor reduction rates (percent reduction in sum of longest diameters were 98, 98, 96, 93, 89, 89, 79, 64, and 62%), accompanied by disappearance of metastases that were present before treatment in 9 patients (Tables 1 and 2; Tables 1 and 2 Supplementary; and Fig. 3). Decrease of plasma CA15-3 levels from start of treatment by 7- to 36-fold occurred in the 6 patients who had elevated marker initially, of whom 4 attained normal levels (Table 2 and Fig. 4). Of 11 patients who were assessed by PET scan, 6 achieved metabolic CRs and 5 patients had metabolic PRs with reduction of SUL_peak_ value by 94, 87, 85, 84, and 50%. Pathologic responses were assessed by mastectomy or by resection with eradication intent in 5 patients. Of three patients who underwent mastectomy, one (Patient 3) with clinical and metabolic CR had pCR (*ypT0N0*), one (Patient 6) with clinical reduction by 96%, and metabolic CR had minimal residual primary staged *ypT1bN0*, and one (Patient 10) with clinical PR (79% reduction) and metabolic PR (85% reduction) had limited residual tumor staged *ypT1cN1a*. Two patients with prior radical mastectomy who attained clinical and metabolic PRs of axillary lymphadenopathy and skin permeation nodules in one (Patient 9), and of permeation nodules in the second (Patient 11), had subsequent resection of soft tissue in the site of mastectomy and lymphadenectomy with eradication intent; small amounts of residual tumor < 1 cm in total diameter were resected in both patients who had experienced disappearance of most tumor targets. In addition, one responder with 98% reduction in tumor diameter and metabolic CR (Patient 4) had no residual breast tumor, and one partial responder (Patient 8) had limited persistent breast tumor infiltration, as assessed by percutaneous biopsy of residual images. Of the 12 patients, 4 had relapsed after progression-free survival (PFS) times of 7, 22, 38, and 51 months, and the other eight had PFS times of 9+, 17+, 21+, 22+, 33+, 44+, 45+, and 55+ months from start of therapy (Table 2, Figs. 3, 5).

### Patients who had not received prior chemotherapy whose tumors overexpressed HER2

Of the 6 patients included in this group, four attained clinical CRs, and 2 had PRs with percent reduction in sum of diameters by 98% in both; responses were accompanied by disappearance of metastases that were present before treatment in 4 patients (Tables 1, 2; Tables 1 and 2 Supplementary; and Fig. 3). Decrease of plasma CA15-3 levels by 2.4- to 518-fold occurred in 3 patients who had elevated markers initially, whose levels became normal in all three (Table 2 and Fig. 4). Of five patients who were assessed by PET scan, 4 attained metabolic CRs and one had metabolic PR with reduction of SUL_peak_ value by 93%. Pathologic response was assessed by mastectomy in 4 clinical complete responders. Pathologic CRs staged *ypT0N0* were attained by 3 patients who had reduction of SUL_peak_ by ≥ 93% (Patients 13–15), and one patient with metabolic CR had residual invasive and intraductal primary staged *ypT1bN0* that did not overexpress HER2 anymore (Patient 16). In addition, one responder with 98% reduction in tumor diameter and metabolic CR (Patient 17) had no residual breast and node tumor as assessed by percutaneous biopsy of residual images. Of the six patients, one had tumor progression after PFS time of 27 months, and the other five had PFS times of 23+, 44+, 67+, 70+, and 71+ months from start of therapy (Table 2, Figs. 3, 5).

### Patients who had received prior chemotherapy

Of nine patients in this group, 8 responded to therapy and one had progressive disease. Of seven responders with clinically measurable disease, 2 attained clinical CRs, and 5 had PRs with tumor reduction in sum of longest diameters by 94, 91, 88, 88, and 81% (Tables 1 and 2; Tables 1 and 2 Supplementary; and Fig. 3). One patient with clinically non-measurable tumor who had only bone metastases experienced dense mineralization of all initially lytic foci, together with metabolic CR (Patient 19). Responses were accompanied by disappearance of most metastases that were present in 8 patients before treatment. Decrease of plasma CA15-3 levels by 2- to 106-fold from baseline occurred in 6 patients who had elevated markers initially, of whom 4 attained normal levels (Fig. 4). Of eight patients who were evaluated by PET scan, 5 attained metabolic CRs and 3 had metabolic PRs with reduction in SUL_peak_ values by 93, 91, and 47%. All responders to therapy had prior mastectomy; none were assessed for pathologic response. Of the eight responders, 6 have relapsed after PFS times of 12, 13, 15, 27, 34, and 54 months from start of therapy, and one patient with prior genotoxic chemotherapy and radiotherapy for childhood’s Ewing’s Tumor, and later for post-operative treatment of breast carcinoma, had fatal AML diagnosed 18 months after completion of the present induction treatment for breast cancer. At diagnosis of AML, the patient had no progression of breast carcinoma; event-free survival (EFS) time was 30 months (Patient 24). One partial responder (Patient 23) had terminated induction treatment with no disease progression; PFS time was 28+ months (Table 2, Figs. 3, 5).

Of twenty-six responders, 23 had clinical evaluation of response (RECIST) together with PET scan assessment (PERCIST). Conformity in percent reduction as assessed by both methods was found in 7 patients (30%) who attained clinical CRs (Fig. 3; Table 2; Tables 1 and 2 Supplementary); moderate disparity in magnitude of response by ≤ 15% reduction rate (mean difference in percent reduction, 6%) was found in 2 patients with clinical CRs and in 13 with clinical PRs (65%); and disparity by 47% reduction was recorded in 1 patient with clinical PR (Patient 22). The difference in the latter consisted in persisting high ^18^FDG uptake in a single bone metastatic site that was mineralized under treatment, while all the other metastatic foci had FDG uptake indistinguishable from surrounding background. Of the three responders who were not evaluated by both methods, one partial responder (Patient 12) had rapidly growing tumor progression preventing further assessment, one metabolic complete responder (Patient 19) had only bone metastasis at presentation that were not measurable, and one complete responder (Patient 18) had PS 4 at presentation preventing completion of initial assessment; the patient had a normal PET scan at the time of final evaluation. All eleven responders who had bone metastases experienced osseous remineralization, and serosal effusion disappeared in 2 patients who had pleura involvement, and in one who had both, pleura, and pericardium involvement. Antitumor responses were rapidly attained (Table 2, and Fig. 5); approximate times required for achieving an objective response from start of treatment ranged from 1.7 to 8.4 months (median, 3.4 months). Responses were accompanied by disappearance of tumor related symptoms in all cases.

Assessment of toxicity before initiation of each cycle of therapy did not record either any form of unusual toxicity or toxic effect of greater magnitude than that expected with each regimen used. Interruption of paclitaxel due to sensory peripheral neuropathy occurred in 12 out of 25 patients (48%) who were treated with the *TCbF* regimen at cumulative amounts of paclitaxel ranging from 156 to 2833 mg/m^2^ (mean, 1534 mg/m^2^), including a single patient who had severe SPN with limb pain, and dysesthesia after the first course of *TCbF*. Patients with paclitaxel induced neuropathy, whose chemotherapy was either interrupted or pursued with *VCbF* had further progressive decrease of neurologic symptoms, and then disappearance occurring in most patients during follow-up. The 13 patients who did not develop SPN under *TCbF* received smaller mean cumulative amount of paclitaxel (mean, 983 mg/m^2^; range 570–1240 mg/m^2^) than that received by patients with neuropathy. The 2 patients whose chemotherapy included *VCbF* without prior paclitaxel did not develop SPN. Except for interruption of paclitaxel as described above, no dose reductions of any cytostatic agent or increasing intervals between courses due to unusual or unexpectedly excessive hematologic and/or visceral toxicity were required. The case of acute myelogenous leukemia carrying the 17q- chromosome aberration marker occurred 18 months after cessation of induction treatment in one patient who had prior genotoxic cytostatics and radiotherapy.

## Discussion

Clinical signification of antitumor potency of chemotherapy regarding long-term outcome for patients with breast carcinoma in advanced stages, an efficiently treatable but essentially incurable condition, is a difficult and amply debated issue. Combination chemotherapy regimens showed a statistically significant advantage over any single agent therapy regarding antitumor response, time to progression and survival^38,39^, but combinations also produce more toxicity leading to detrimental effects on quality of life. Moreover, for patients unselected for phenotypic sub specificities, there is no recognized standard combination regimen among the most active ones, since taxane-containing combinations were significantly but only modestly better than anthracycline-based combinations in terms of response rate and PFS, but not for survival^39^. Among combination treatments, platinum derivative-containing regimens of various compositions were reported to be slightly more potent than non-platinum combination treatments regarding response rate and event-free survival, this statistically significant difference being more marked in the subset of patients with triple negative advanced breast carcinoma in which moderate improvement of survival was also found^56^. Remarkably, improvement of long-term prognosis related to degree of antitumor efficacy of induction treatments has been firmly demonstrated from studies of patients with high-risk localized breast carcinoma subjected to pre-operative induction treatment. Meta-analysis of a large number of studies demonstrated that patients who attained pathologic complete responses under preoperative treatment had much greater event-free and survival times than did those who had residual tumor, these statistically significant improvements being maintained over long periods of time^57^. However, pathologic CRs, whose rate vary in subsets of patients with phenotypically distinct tumors, are attained by only approximately 20% of all patients with clinically localized breast cancer subjected to induction treatment^57^, which emphasizes the need for powerful newer strategies applicable to all subgroups of patients with this neoplasm in need of chemotherapy.

In an attempt at improvement of the antitumor potency of chemotherapy, we explored a new method to modulate the cytotoxic activity of FUra and folinic acid included in standard regimens by adding high-dose pyridoxine accompanying each administration of FUra and FA. Response to therapy was rapidly attained by 26 out of 27 patients included in the three subsets presented herein. However, neither this strikingly high response rate nor the short median time of 3.4 months required to attain a response can be paired to prior therapeutic series for a comparison. Antitumor responses of great magnitude were attained by all eighteen previously untreated patients presenting with unresectable breast adenocarcinoma, of which most had numerous metastases (Table 2). Complete responses of long duration and partial responses with great tumor reduction rates were achieved by these patients, with the more marked favorable overall results attained by patients whose tumors overexpressed HER2, as expected from prior studies with patients treated with chemotherapy associated with anti HER-2/neu monoclonal antibodies, preferably with trastuzumab and pertuzumab combined^58^. Moreover, in 6 of 12 previously untreated patients who attained a clinical response with reduction in sum of tumor diameters by 96% or more who were subjected to mastectomy (Table 2, Figs. 3, 5), two had only small residual tumor staged *ypT1bN0*, and 4 had pathologic CRs. In addition, 2 other responders with tumor reduction rate of 98% in both had no residual tumor in breast as assessed by ultrasonography-oriented biopsy of images persisting after induction treatment. Although still partial, these results outline the capacity of the treatment presented herein at achieving major tumor reduction, and eventually disease eradication in patients with previously untreated advanced inoperable and/or metastatic breast carcinoma expressing or not the ERs, either without or with HER2 overexpression. Complete clinical and metabolic responses of long duration and partial responses with great tumor reduction rates together with disappearance of metastases were also achieved by patients who had received prior treatment. Among the 8 responders, 7 had been treated previously with combination chemotherapy regimens including FUra, of whom 4 received folinic acid as well. Further studies are needed to explore whether addition to FUra of folinic acid and high-dose B6 vitamer in tandem can overcome prior resistance to the fluoropyrimidines. The present PN dose escalation pilot study does not enable correlating the magnitude of antitumor responses nor the rapidity to attain a response with the median daily dose of B6 vitamer received by each patient during the time of treatment (Table 2). Clinical trials including pharmacokinetically-monitored dose finding studies are necessary to explore this issue.

Assessment of toxicity due to treatment before initiation of each cycle could not discover any form of toxicity greater than that expected with each regimen in the absence of B6 vitamer, or any unexpected toxic effect. In particular, the use of high cumulative doses of pyridoxine was not accompanied with greater incidence, or higher grades, of sensory peripheral neuropathy than that expected with the use of paclitaxel as scheduled in the *TCbF* regimen. Except for one patient who had early onset acute sensory peripheral neuropathy following the first course of *TCbF*, the cumulative amount of paclitaxel received by patients who experienced dose-limiting sensory peripheral neuropathy was within the range of that previously reported^59^.

The great magnitude of antitumor responses which were rapidly attained by patients with breast carcinoma who carried great tumor burden in most cases, suggest that addition of vitamin B6 in high dose strongly enhances the antitumor activity of combination regimens comprising FUra and FA. This antitumor potency may predict for favorable long-term outcomes as reported from studies of patients with colorectal carcinoma^60^, and breast carcinoma^61^ in advanced stages that attained early tumor shrinkage and deep antitumor responses under induction treatment.

The remarkable antitumor activity observed in the present pilot study may represent the difference with that reported elsewhere in trials using combination regimens administered in their standard form. Strength of antitumor activity achieved in the present study is of similar level than that recently reported in patients with advanced carcinomas of the digestive tract treated with regimens including FUra, FA, and PN^52^. Murine experiments reported herein indicate that parenteral PM carries an advantage over PN to expand intracellular PLP pools which may facilitate SHMT-dependent synthesis of CH_2_-H_4_PteGlu to improve the modulation of FUra. Exploration of these findings requires first the production and development of pyridoxamine for clinical use.

Demonstration of potentiation of FUra by FA and high dose B6 vitamer in tandem requires clinical trials of combination schemas for patients with potentially FUra-sensitive tumors. Vitamin B6 pharmacokinetics studies with emphasis on intracellular PLP levels^29,30,33,35,62^, should accompany these trials to optimize the modulation of fluoropyrimidines in accordance with experimental data.

## Supplementary Information

Supplementary Table 1.

Supplementary Table 2.

## Data Availability

We state that all data generated during this study are included in the article. Data and materials are reported in the text under Materials and Methods, and Results sections, in Tables 1 and 2, and in Supplementary Tables 1, and 2.

## References

[1] Santi DV, McHenry CS, Sommer H (1974). Mechanism of interaction of thymidylate synthetase with 5-fluorodeoxyuridylate. Biochemistry.

[2] Danenberg PV, Danenberg KD (1978). Effect of 5,10-methylenetetrahydrofolate on the dissociation of 5-Fluoro-2’-deoxyuridylate from thymidylate synthetase: Evidence for an ordered mechanism. Biochemistry.

[3] Lockshin A, Danenberg PV (1981). Biochemical factors affecting the tightness of fluorodeoxyuridylate binding to human thymidylate synthetase. Biochem. Pharmacol..

[4] Ullman B, Lee M, Martin DW, Santi DV (1978). Cytotoxicity of 5-fluoro-2'-deoxyuridine: Requirement for reduced folate cofactors and antagonism by methotrexate. Proc. Natl. Acad. Sci. USA.

[5] Wyatt MD, Wilson DM (2009). Participation of DNA repair in the response to 5-fluorouracil. Cell Mol. Life Sci..

[6] Mani C, Pai S, Papke CM, Palle K, Gmeiner WH (2018). Thymineless death by the fluoropyrimidine polymer F10 involves replication fork collapse and is enhanced by Chk1 inhibition. Neoplasia.

[7] Cortez D (2015). Preventing replication fork collapse to maintain genome integrity. DNA Repair.

[8] Hagenkort A (2017). dUTPase inhibition augments replication defects of 5-Fluorouracil. Oncotarget.

[9] Machover D (1982). Treatment of advanced colorectal and gastric adenocarcinomas with 5-FU combined with high-dose folinic acid: A pilot study. Cancer Treat. Rep..

[10] Piedbois P (1992). Modulation of 5-fluorouracil by leucovorin in patients with advanced colorectal cancer: Evidence in terms of response rate. J. Clin. Oncol..

[11] Machover D (1993). 5-FluorouraciI combined with the [6S]-stereoisomer of folinic acid in high doses for treatment of patients with advanced colorectal carcinoma. A phase I-II study of two consecutive regimens. Ann. Oncol..

[12] André T (2007). Phase III study comparing a semimonthly with a monthly regimen of fluorouracil and leucovorin as adjuvant treatment for stage II and III colon cancer patients: Final results of GERCOR C96.1. J. Clin. Oncol..

[13] Romanini A (1991). Role of folylpolyglutamates in biochemical modulation of fluoropyrimidines by leucovorin. Cancer Res..

[14] Wright JE (1989). Selective expansion of 5,10-Methylenetetrahydrofolate pools and modulation of 5-fluorouracil antitumor activity by leucovorin in vivo. Cancer Res..

[15] Houghton JA (1990). Influence of dose of [6*RS*]Leucovorin on reduced folate pools and 5-fluorouracil-mediated thymidylate synthase inhibition in human colon adenocarcinoma xenografts. Cancer Res..

[16] Houghton JA (1990). Relationship between dose rate of [6*RS*]Leucovorin administration, plasma concentrations of reduced folates, and pools of 5,10-methylenetetrahydrofolates and tetrahydrofolates in human colon adenocarcinoma xenografts. Cancer Res..

[17] Zhang ZG, Rustum YM (1991). Effect of diastereoisomers of 5-formyltetrahydrofolate on cellular growth, sensitivity to 5-fluoro-2’-deoxyuridine, and methylenetetrahydrofolate polyglutamate levels in HCT-8 cells. Cancer Res..

[18] Boarman DM, Allegra CJ (1992). Intracellular metabolism of 5-formyl tetrahydrofolate in human breast and colon cell lines. Cancer Res..

[19] Priest DG, Schmitz JC, Bunni MA, Ayling JE (1993). Folate metabolites as modulators of antitumor drug activity. Chemistry and Biology of Pteridines and Folates.

[20] Voeller DM, Allegra CJ (1994). Intracellular metabolism of 5-methyltetrahydrofolate and 5-formyltetrahydrofolate in a human breast cancer cell line. Cancer Chemother. Pharmacol..

[21] Machover D (2001). Cytotoxic synergism of methioninase in combination with 5-fluorouracil and folinic acid. Biochem. Pharmacol..

[22] Wettergren Y, Taflin H, Odin E, Kodeda K, Derwinger K (2015). A pharmacokinetic and pharmacodynamic investigation of Modufolin compared to Isovorin after single dose intravenous administration to patients with colon cancer: A randomized study. Cancer Chemother. Pharmacol..

[23] Nixon PF, Slutsky G, Nahas A, Bertino JR (1973). The turnover of folate coenzymes in murine lymphoma cells. J. Biol. Chem..

[24] Schirch LV (1973). Serine transhydroxymethylase. Subunit structure and the involvement of sulfhydryl groups in the activity of the enzyme. J. Biol. Chem..

[25] Jones CW, Priest DG (1978). Interaction of pyridoxal 5’-phosphate with apo-serine hydroxymethyl transferase. Biochim. Biophys. Acta.

[26] Perry C, Yu S, Chen J, Matharu KS, Stover PJ (2007). Effect of vitamin B6 availability on serine hydroxymethyltransferase in MCF-7 cells. Arch. Biochem. Biophys..

[27] Giardina G (2015). How pyridoxal 5’-phosphate differentially regulates human cytosolic and mitochondrial serine hydroxymethyltransferase oligomeric state. FEBS J..

[28] Kikuchi G, Motokawa Y, Yoshida T, Hiraga K (2008). Glycine cleavage system: Reaction mechanism, physiological significance, and hyperglycinemia. Proc. Jpn. Acad. Ser. B Phys. Biol. Sci..

[29] Ueland PM, Ulvik A, Rios Ávila L, Midttun Ø, Gregory JF (2015). Direct and functional biomarkers of vitamin B6 status. Annu. Rev. Nutr..

[30] Zempleni J, Kübler W (1994). The utilization of intravenously infused pyridoxine in humans. Clin. Chim. Acta.

[31] Martínez M, Cuskelly GJ, Williamson J, Toth JP, Gregory JF (2000). Vitamin B-6 deficiency in rats reduces hepatic serine hydroxymethyl transferase and cystathionine β-synthase activities and rates of in vivo protein turnover, homocysteine remethylation and transsulfuration. J. Nutr..

[32] Scheer JB, Mackey AD, Gregory JF (2005). Activities of hepatic cytosolic and mitochondrial forms of serine hydroxymethyltransferase and hepatic glycine concentration are affected by vitamin B-6 intake in rats. J. Nutr..

[33] Machover D (2018). Enhancement of 5-fluorouracil cytotoxicity by pyridoxal 5'-phosphate and folinic acid in tandem. J. Pharmacol. Exp. Ther..

[34] Beechey RP, Happold FC (1957). Pyridoxamine phosphate transaminase. Biochem. J..

[35] Ink SL, Henderson LM (1984). Vitamin B6 metabolism. Ann. Rev. Nutr..

[36] Di Salvo ML, Contestabile R, Safo MK (2011). Vitamin B6 salvage enzymes: Mechanism, structure and regulation. Biochim. Biophys. Acta.

[37] Musayev FN, Di Salvo ML, Ko TP, Schirch V, Safo MK (2003). Structure and properties of recombinant human pyridoxine 5′-phosphate oxidase. Protein Sci..

[38] Carrick S (2009). Single agent versus combination chemotherapy for metastatic breast cancer. Cochrane Database Syst. Rev..

[39] Piccart-Gebhart MJ (2008). Taxanes alone or in combination with anthracyclines as first-line therapy of patients with metastatic breast cancer. J. Clin. Oncol..

[40] Swain SM (1989). Fluorouracil and high dose leucovorin in previously treated patients with metastatic breast cancer. J. Clin. Oncol..

[41] Margolin KA (1992). Effective initial therapy of advanced breast cancer with fluorouracil and high-dose, continuous infusion calcium leucovorin. J. Clin. Oncol..

[42] Zaniboni A (1993). L-folinic acid and 5-fluorouracil in the treatment of advanced breast cancer: A phase II study. Ann. Oncol..

[43] Nolè F (1997). Phase I-II study of vinorelbine in combination with 5-fluorouracil and folinic acid as first-line chemotherapy in metastatic breast cancer: A regimen with a low subjective toxic burden. Ann. Oncol..

[44] Kornek GV (1998). Effective treatment of advanced breast cancer with vinorelbine, 5-fluorouracil and l-leucovorin plus human granulocyte colony-stimulating factor. Br. J. Cancer.

[45] Gebbia V (2006). Vinorelbine and 5-fluorouracil bolus and/or continuous venous infusion plus levofolinic acid as second-line chemotherapy for metastatic breast cancer: An analysis of results in clinical practice of the Gruppo Oncologico Italia Meridionale (GOIM). Anticancer Res..

[46] Nicholson BP (2000). Paclitaxel, 5-fluorouracil, and leucovorin (TFL) in the treatment of metastatic breast cancer. Clin. Breast Cancer.

[47] Loesch DM (2003). A phase II trial of weekly paclitaxel, 5-fluorouracil, and leucovorin as first-line treatment for metastatic breast cancer. Breast Cancer Res. Treat..

[48] O'Shaughnessy JA (1994). A dose intensity study of FLAC (5-fluorouracil, leucovorin, doxorubicin, cyclophosphamide) chemotherapy and *Escherichia coli*-derived granulocyte macrophage colony-stimulating factor (GM-CSF) in advanced breast cancer patients. Ann. Oncol..

[49] Colucci G (1995). Laevofolinic acid, 5-fluorouracil, cyclophosphamide, and escalating doses of epirubicin with granulocyte colony-stimulating factor support in locally advanced and/or metastatic breast carcinoma: A phase I-II study of the Southern Italy Oncology Group (GOIM). Br. J. Cancer.

[50] Schaumburg H (1983). Sensory neuropathy from pyridoxine abuse. A new megavitamin syndrome. N. Engl. J. Med..

[51] Rimland B, Callaway E, Dreyfus P (1978). The effect of high doses of vitamin B6 on autistic children: A double blind crossover study. Am. J. Psychiatry.

[52] Machover D (2021). Pharmacologic modulation of 5-fluorouracil by folinic acid and high-dose pyridoxine for treatment of patients with digestive tract carcinomas. Sci. Rep..

[53] Freireich EJ, Gehan EA, Rall DP, Schmidt LH, Skipper HE (1966). Quantitative comparison of toxicity of anticancer agents in mouse, rat, hamster, dog, monkey, and man. Cancer Chemother. Rep..

[54] Eisenhauer EA (2009). New response evaluation criteria in solid tumours: Revised RECIST guideline (version 1.1). Eur. J. Cancer..

[55] Pinker K, Riedl C, Weber WA (2017). Evaluating tumor response with FDG PET: Updates on PERCIST, comparison with EORTC criteria and clues to future developments. Eur. J. Nucl. Mol. Imaging.

[56] Egger SJ (2017). Platinum-containing regimens for metastatic breast cancer. Cochrane Database Syst. Rev..

[57] Spring LM (2020). Pathological complete response after neoadjuvant chemotherapy and impact on breast cancer recurrence and survival: A comprehensive meta-analysis. Clin. Cancer Res..

[58] Baselga J, for the CLEOPATRA Study Group (2012). Pertuzumab plus trastuzumab plus docetaxel for metastatic breast cancer. N. Engl. J. Med..

[59] Miltenburg NC, Boogerd W (2014). Chemotherapy-induced neuropathy: A comprehensive survey. Cancer Treat. Rev..

[60] Taïeb J (2018). Exploratory analyses assessing the impact of early tumour shrinkage and depth of response on survival outcomes in patients with RAS wild-type metastatic colorectal cancer receiving treatment in three randomised panitumumab trials. J. Cancer Res. Clin. Oncol..

[61] Che Y-Q (2020). Depth of response and early tumor shrinkage for predicting clinical outcomes in HER2-positive metastatic breast cancer treated with trastuzumab. Cancer Manag. Res..

[62] Mehansho H, Henderson LM (1980). Transport and accumulation of pyridoxine and pyridoxal by erythrocytes. J. Biol. Chem..

