# Supplementary material for: Pharmacologic modulation of 5-fluorouracil by folinic acid and pyridoxine for treatment of patients with advanced breast carcinoma
Source: Sci Rep. 2022 May 31;12:9079. doi: 10.1038/s41598-022-12998-5 (PMC9156777; doi:10.1038/s41598-022-12998-5)
Supplement: Supplementary file 1 — Supplementary Table 1. [file 41598_2022_12998_MOESM1_ESM.pdf]

Table 1 Supplementary. Selected CT scan and MR imaging from patients with advanced breast carcinoma who responded to regimens comprising FUra, folinic acid and pyridoxine in tandem

Patient no.	Before treatment	After treatment
1	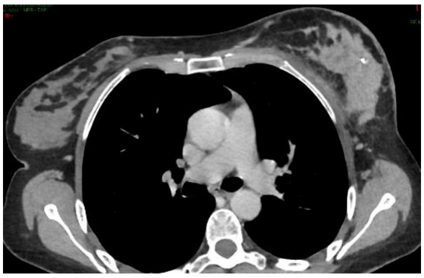	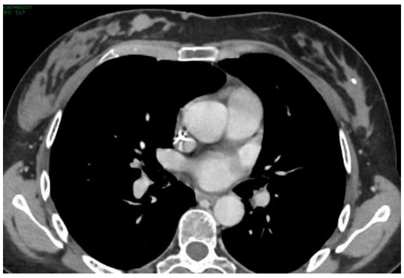
2	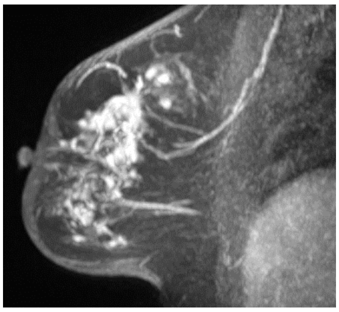	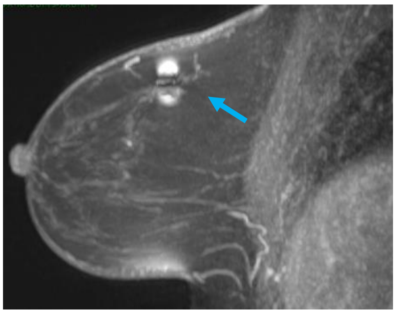
3	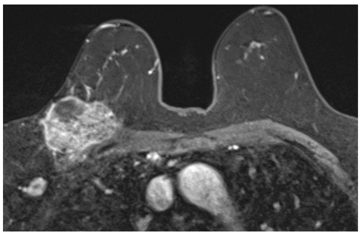	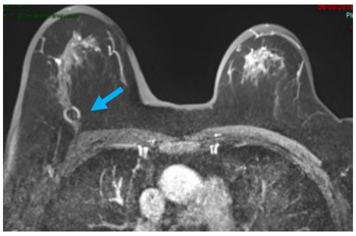
4	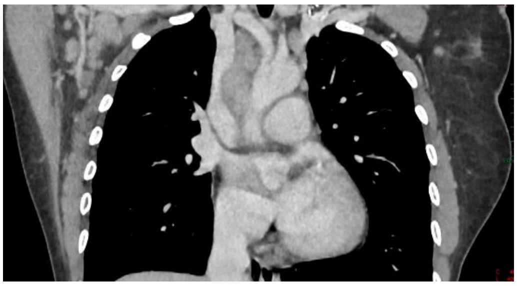	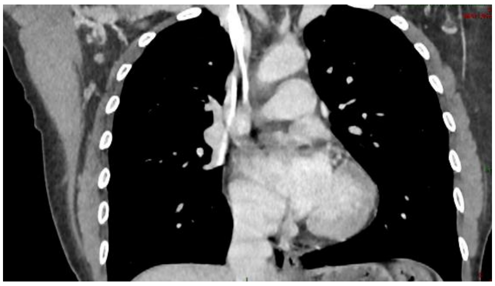
5	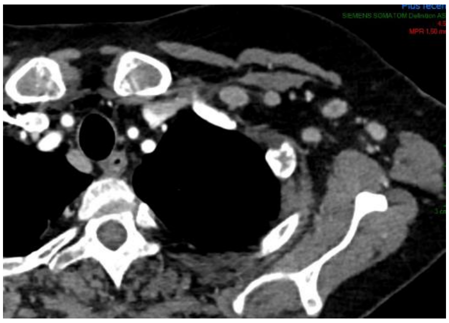	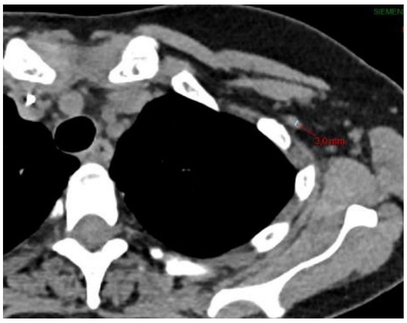
6	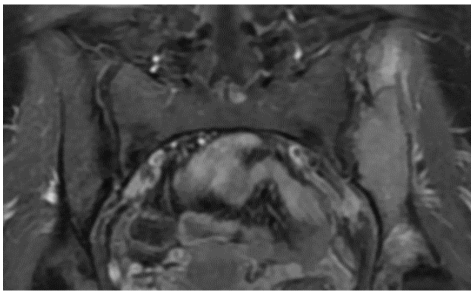	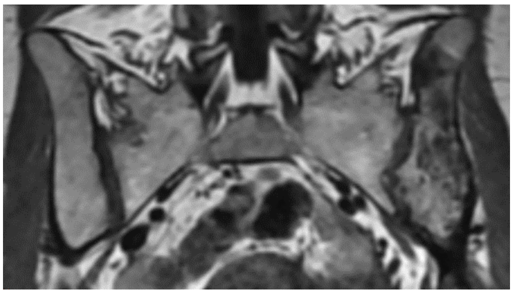

**7[1]**	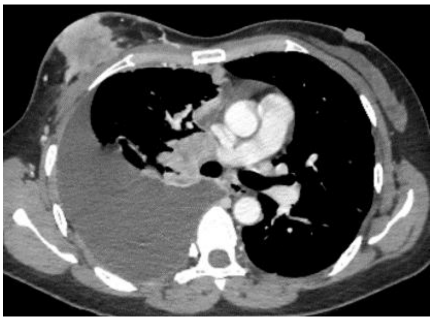	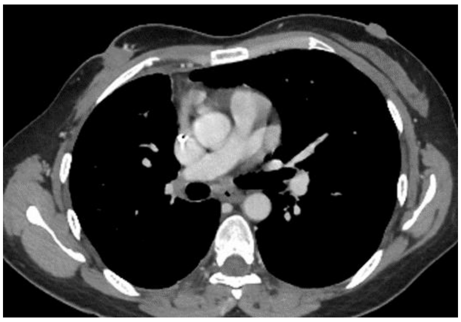
**8**	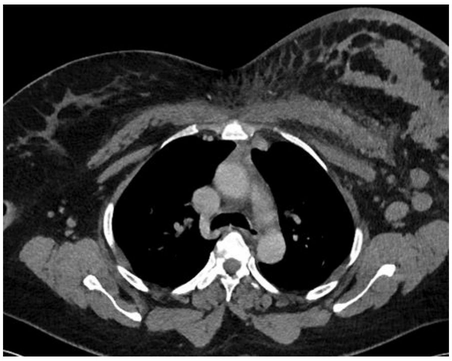	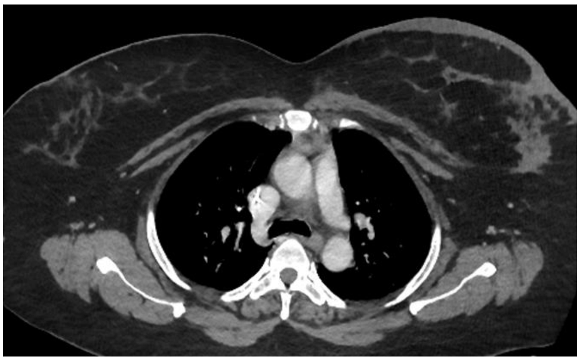
**9**	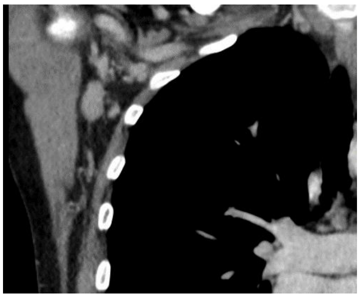	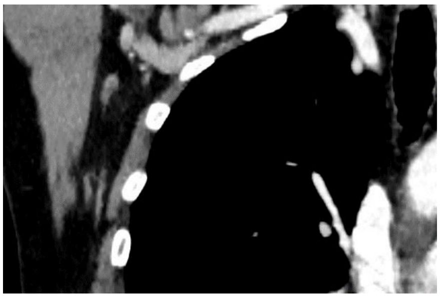
**10**	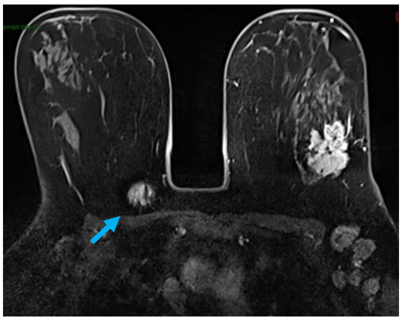	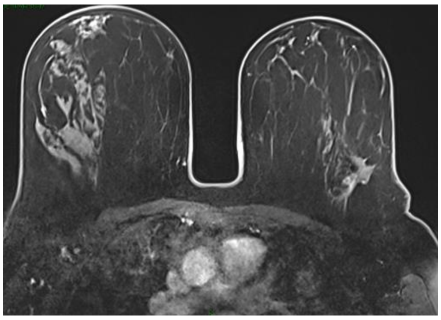
**12**	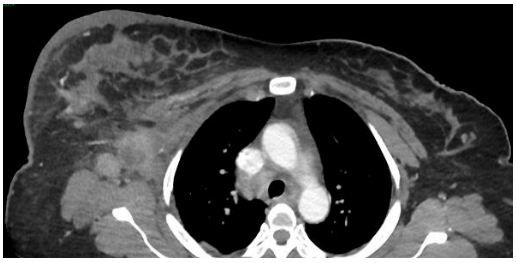	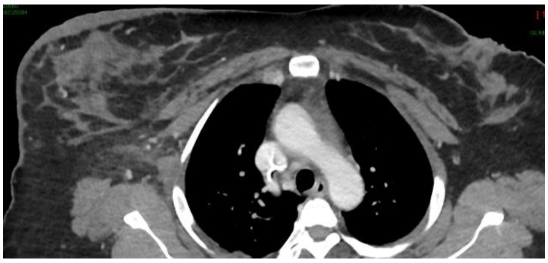
**13**	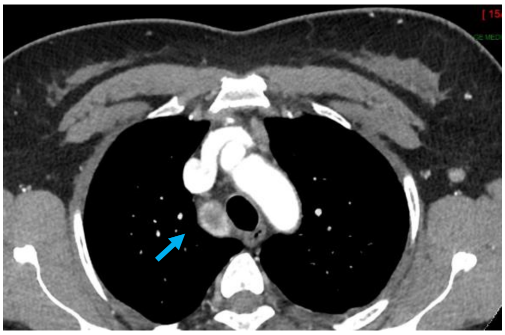	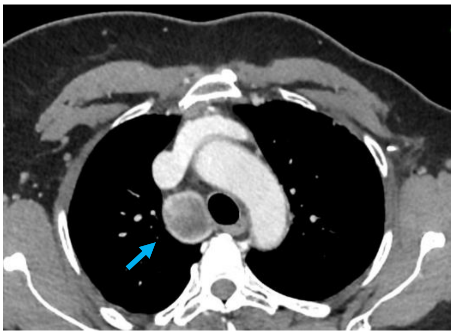

**14**	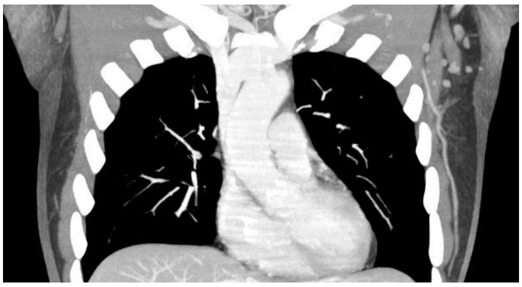	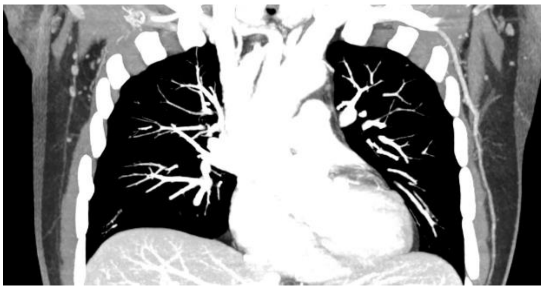
**15**	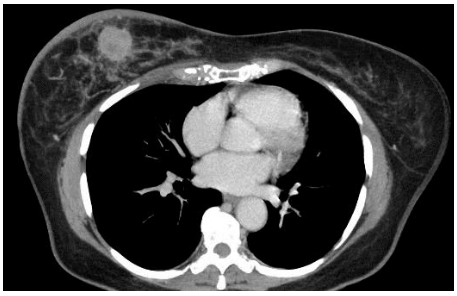	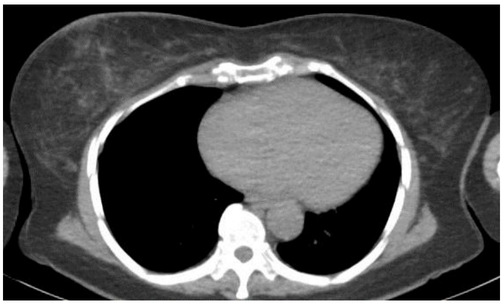
**16**	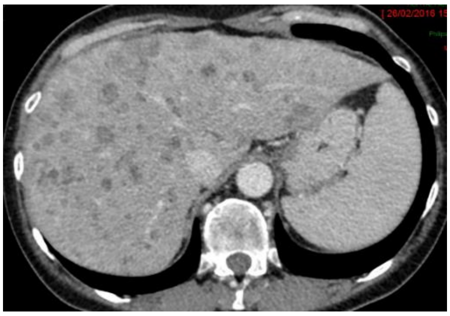	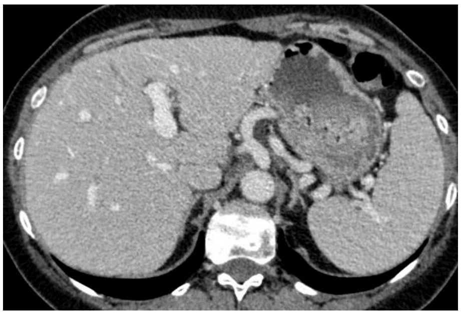
**17**	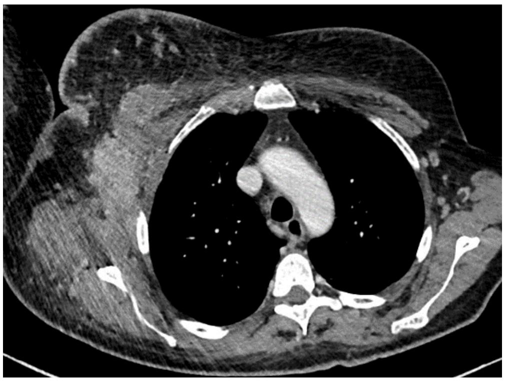	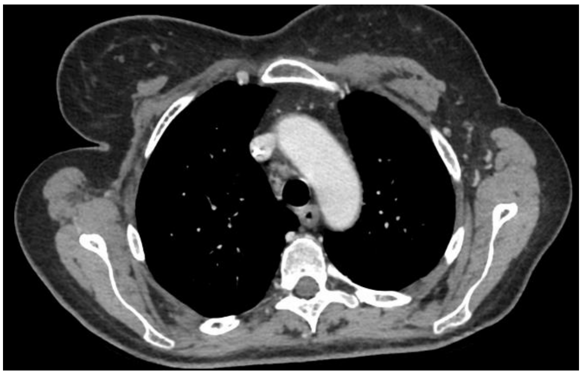
**18**	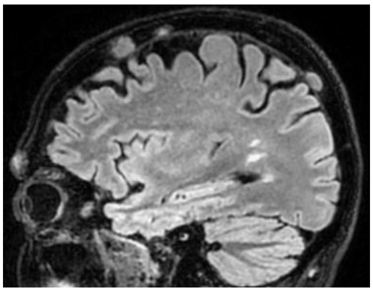	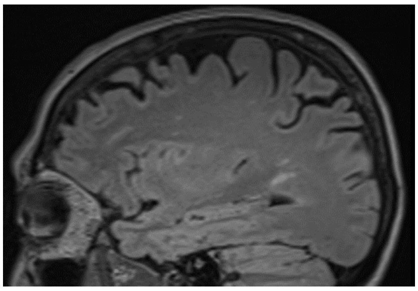
**19**	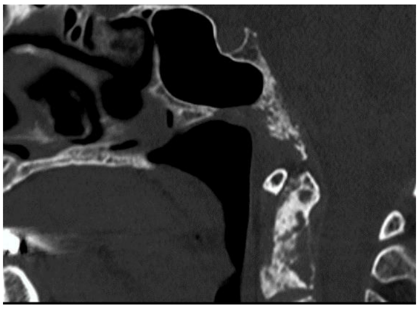	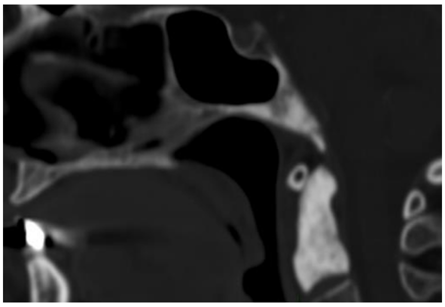

**20**	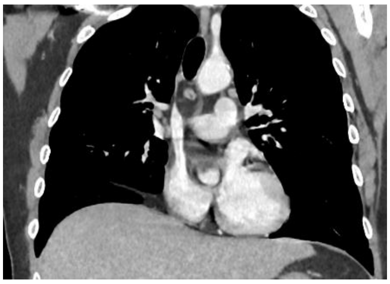	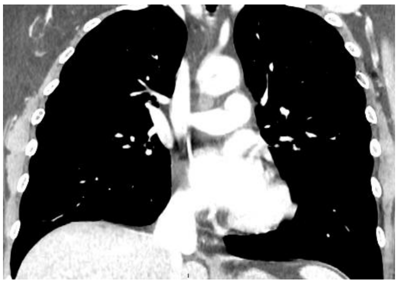
**21**	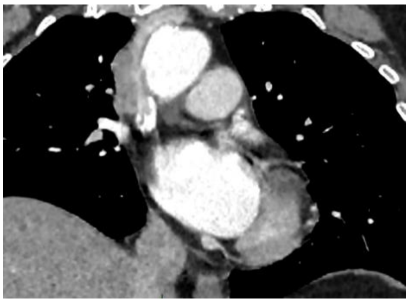	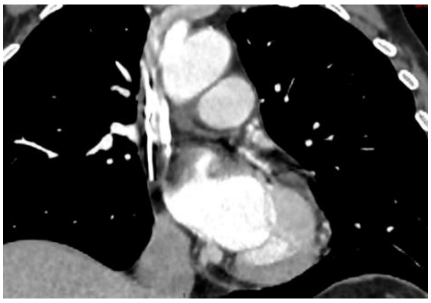
**22**	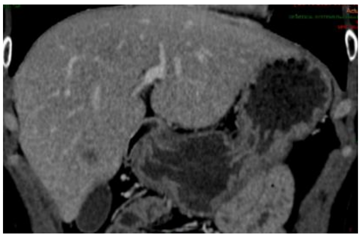	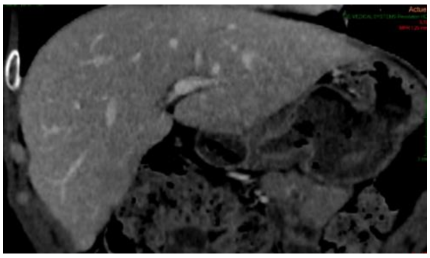
**23**	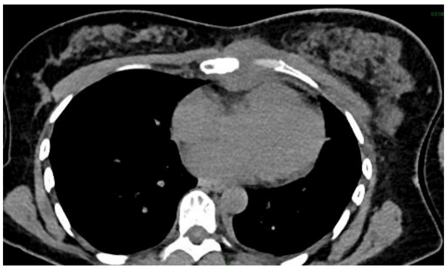	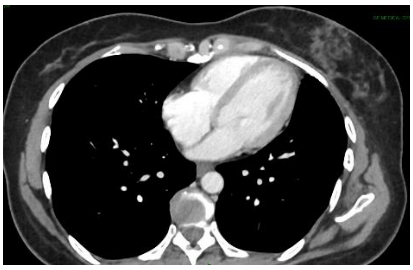
**24**	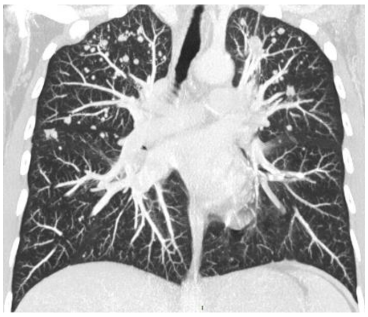	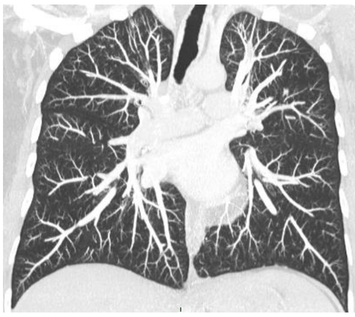
**25**	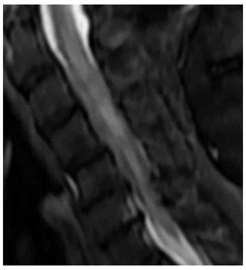	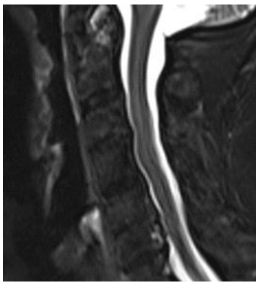

**26**	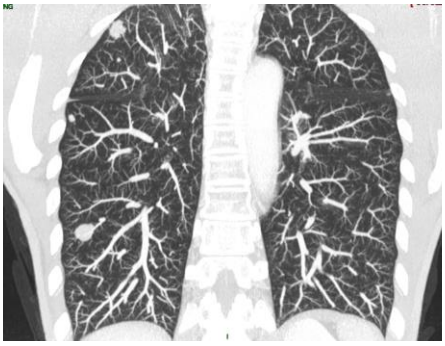	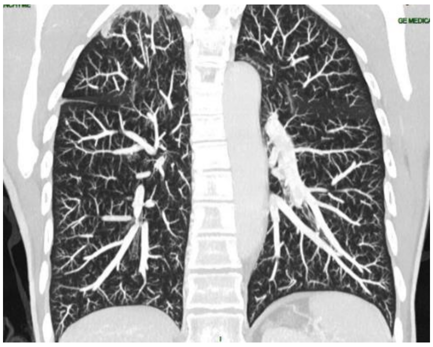

Patients are numbered in the order of that in Tables 1, and 2, and Figures 3, and 5. Iconography of patient 11 who had exclusive skin permeation nodules was only represented in Table 2 Supplementary. Blue arrows indicate non-tumor images: Patients 2, 3, and 10, artifacts; Patient 13, thoracic goiter. Selected tumor-related images in the present Table are indicated with red arrows in Table 2 Supplementary. [1]Patient whose induction treatment was still ongoing at the time of present assessment.
