# Supplementary material for: Pharmacologic modulation of 5-fluorouracil by folinic acid and pyridoxine for treatment of patients with advanced breast carcinoma
Source: Sci Rep. 2022 May 31;12:9079. doi: 10.1038/s41598-022-12998-5 (PMC9156777; doi:10.1038/s41598-022-12998-5)
Supplement: Supplementary file 2 — Supplementary Table 2. [file 41598_2022_12998_MOESM2_ESM.pdf]

Table 2 Supplementary. Selected ^{18}FDG PET Scan imaging from patients with advanced breast carcinoma who responded to regimens comprising FUra, folinic acid and pyridoxine in tandem

Patient no.	Before treatment	After treatment
1	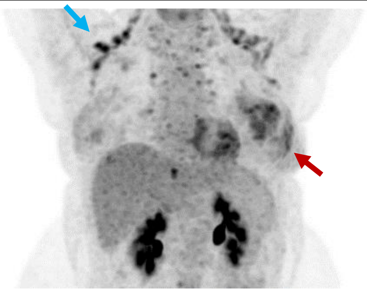	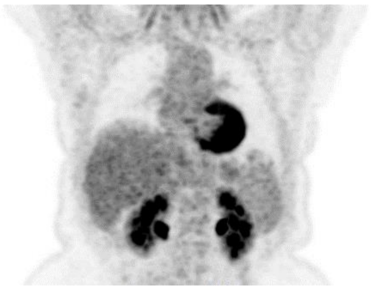
2	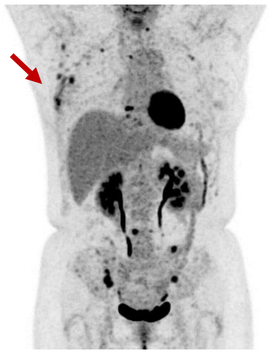	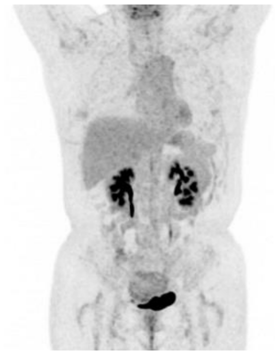
3	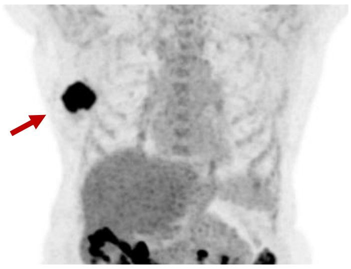	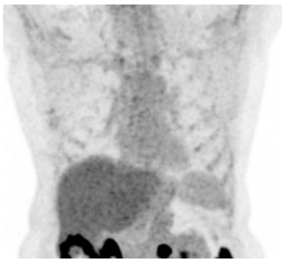
4	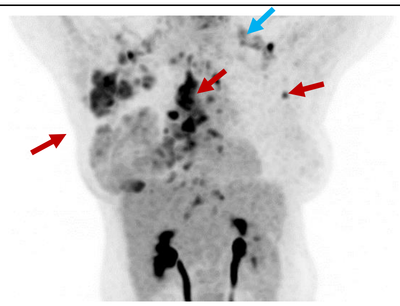	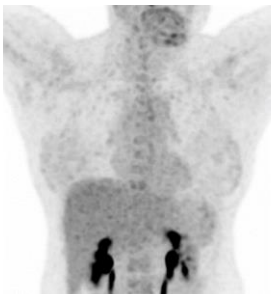
5	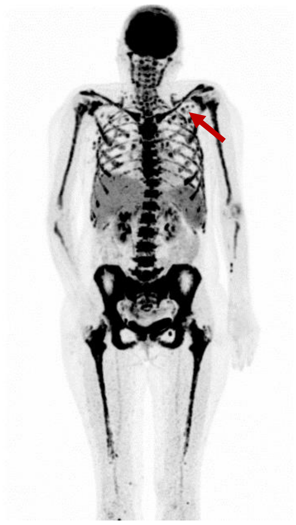	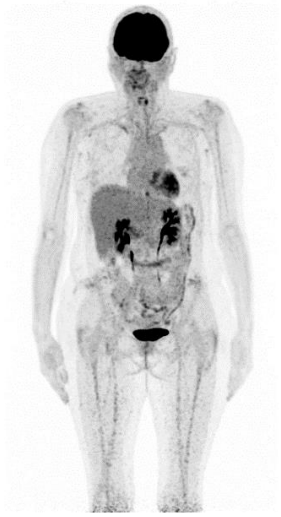

**6**	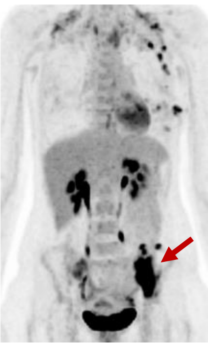	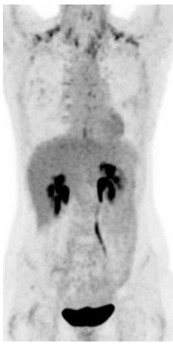
**7[1]**	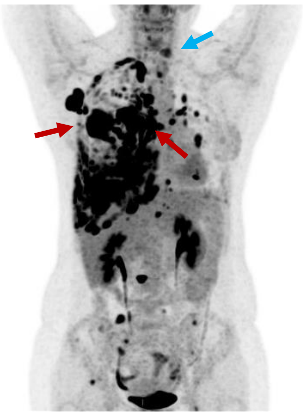	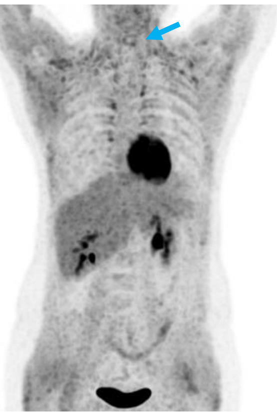
**8**	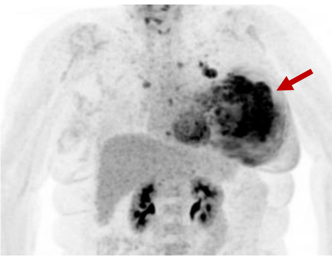	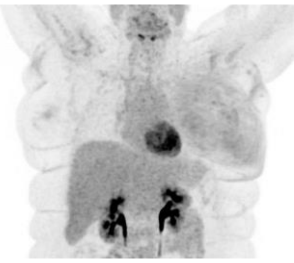
**9**	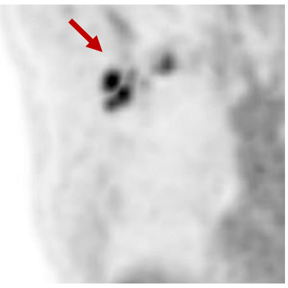	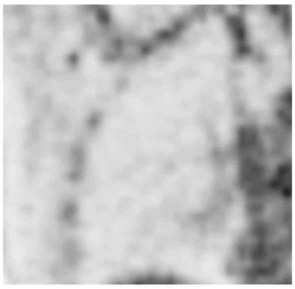
**10**	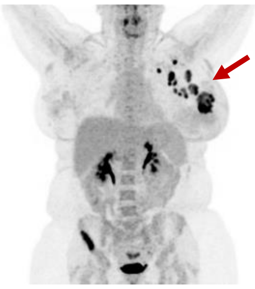	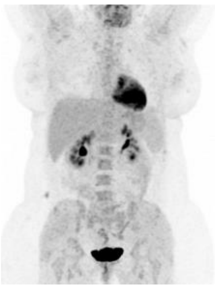
**11**	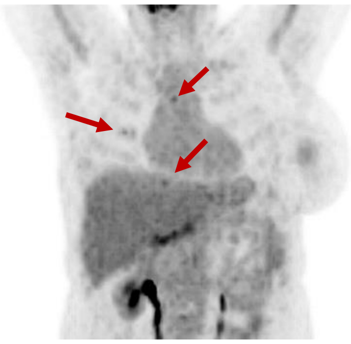	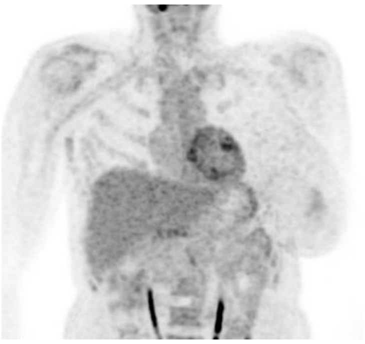

**13**	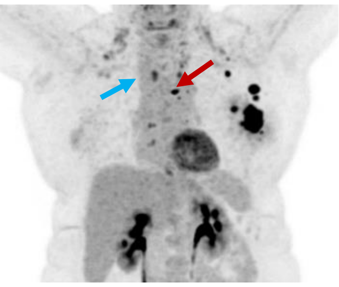	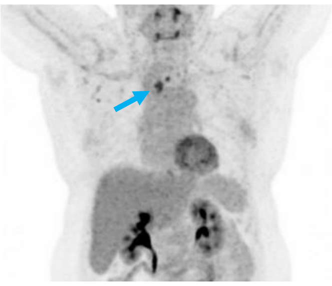
**14**	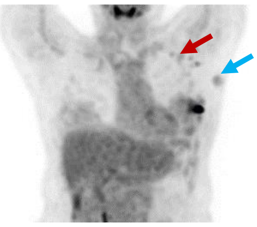	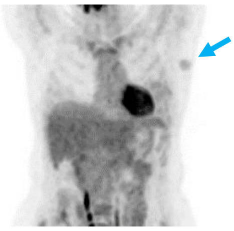
**15**	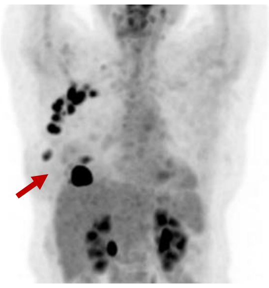	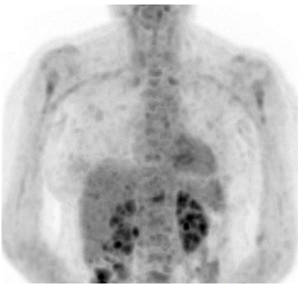
**16**	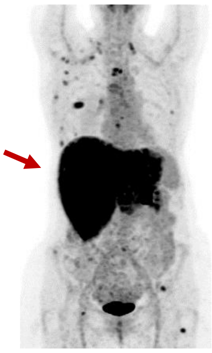	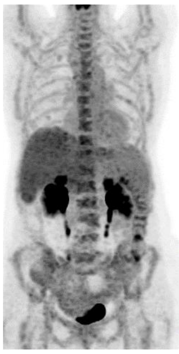
**17**	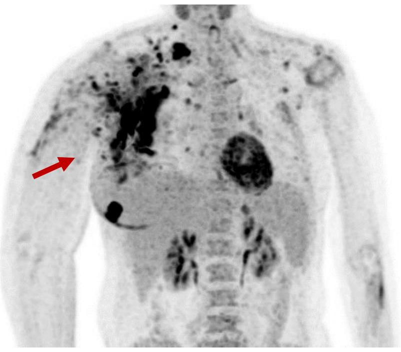	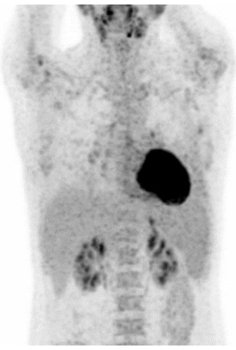
**19**	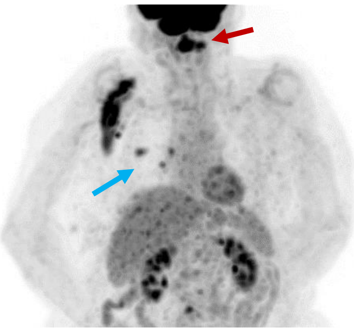	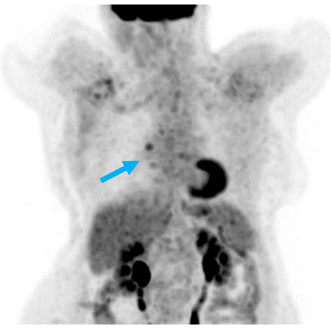

**20**	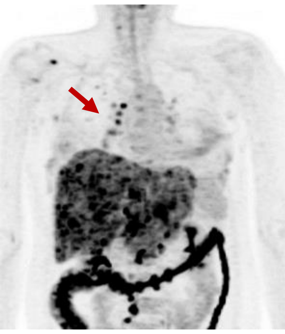	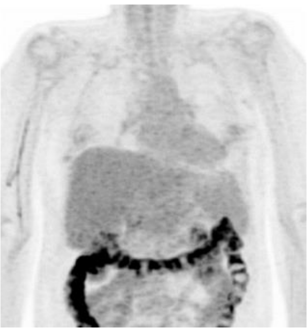
**21**	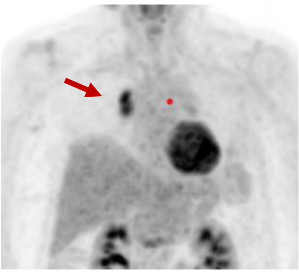	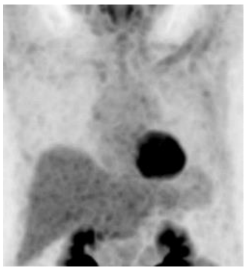
**22**	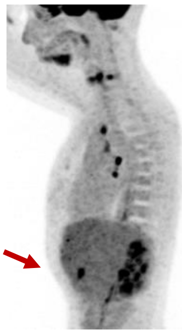	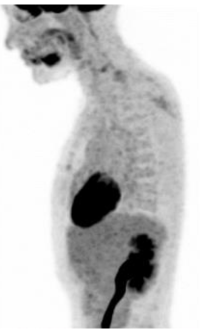
**23**	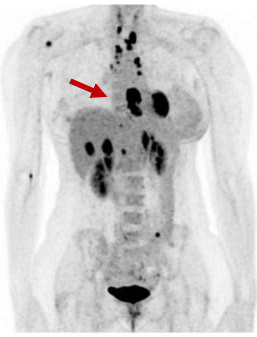	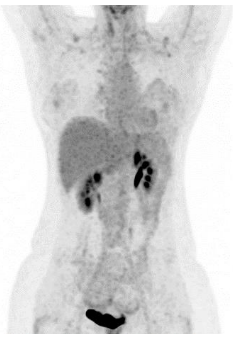
**24**	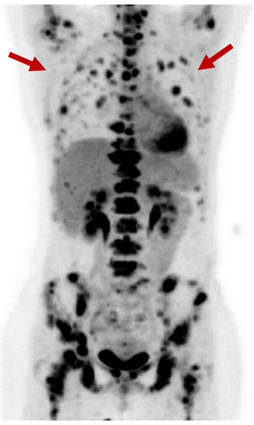	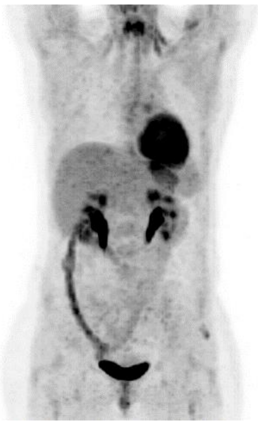
**25**	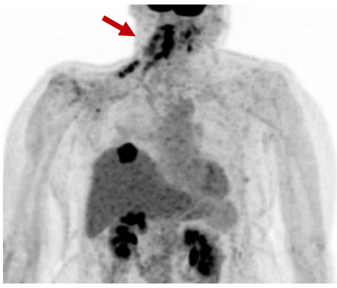	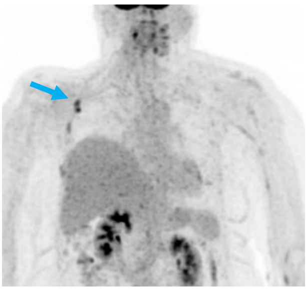

**26**	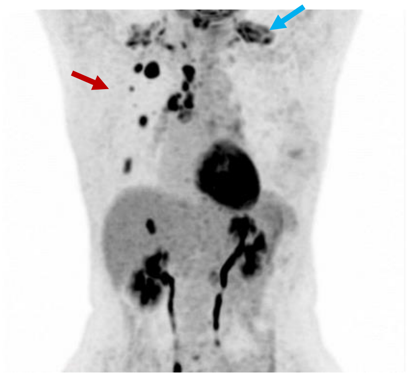	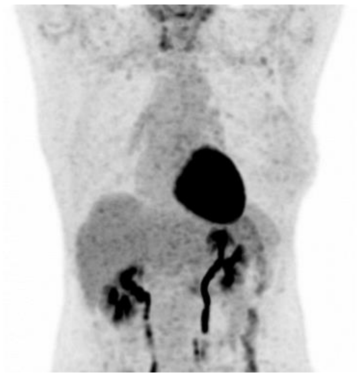

Patients are numbered in the order of that in Tables 1, and 2, and Figures 3, and 5. Two patients (12 and 18) are not represented owing to absence of final (Patient 12), or initial (Patient 18) PET Scan assessment (see text). Blue arrows indicate non-tumor images; these are: Patients 1, 4, and 26, brown fat; Patient 7, goiter and brown fat; Patient 13, intrathoracic goiter; Patient 19, mycobacterium pneumonitis treated successfully; Patient 25 right panel, rib trauma; Patient 14, non-tumor high focal skin FDG uptake of undetermined significance. Red arrows indicate selected tumor-related images that are represented in Table 1 Supplementary, and skin permeation nodules in Patient 11. [1]Patient whose induction treatment was ongoing at the time of present assessment.
